# Integrating advanced deep learning techniques for enhanced detection and classification of citrus leaf and fruit diseases

**DOI:** 10.1038/s41598-025-97159-0

**Published:** 2025-04-12

**Authors:** Archna Goyal, Kamlesh Lakhwani

**Affiliations:** Department of Computer Science and Engineering, JECRC University, Jaipur, 303905 Rajsthan India

**Keywords:** Citrus diseases, Deep learning, InceptionV3, DenseNet121, Agricultural disease management, Plant sciences, Diseases

## Abstract

In this study, we evaluate the performance of four deep learning models, EfficientNetB0, ResNet50, DenseNet121, and InceptionV3, for the classification of citrus diseases from images. Extensive experiments were conducted on a dataset of 759 images distributed across 9 disease classes, including Black spot, Canker, Greening, Scab, Melanose, and healthy examples of fruits and leaves. Both InceptionV3 and DenseNet121 achieved a test accuracy of 99.12%, with a macro average F1-score of approximately 0.986 and a weighted average F1-score of 0.991, indicating exceptional performance in terms of precision and recall across the majority of the classes. ResNet50 and EfficientNetB0 attained test accuracies of 84.58% and 80.18%, respectively, reflecting moderate performance in comparison. These research results underscore the promise of modern convolutional neural networks for accurate and timely detection of citrus diseases, thereby providing effective tools for farmers and agricultural professionals to implement proactive disease management, reduce crop losses, and improve yield quality.

## Introduction

Oranges, lemons, limes, grapefruits and tangerines are among the most cultivated and economically most significant crops in the world. In 2020, global citrus production reached approximately 158 million metric tons, with oranges alone accounting for over 73 million metric tons^1^. Citrus crops are a vital source of nutrition, providing essential vitamins and minerals, and play a crucial role in the economies of many countries, supporting the livelihoods of millions of people involved in cultivation, processing, and distribution^1,2^. The global citrus industry, however, faces substantial challenges due to various diseases and disorders affecting leaves and fruits, which can lead to significant yield losses and quality degradation^3,4^. Among these diseases, Huanglongbing (HLB), also known as citrus greening disease, is considered the most devastating, causing up to 100% tree mortality in severely affected areas^5^. In Brazil and the United States, two of the largest citrus producers, HLB has led to drastic reductions in citrus production over the past decade^6,7^. If implemented in a timely manner, these are crucial diseases to facilitate early detection and accurate classification for mitigating the economic losses. For instance, effective management of HLB requires the prompt removal of infected trees and control of the vector insect *Diaphorina citri*^8^. Delayed or inaccurate detection can result in rapid disease spread, exacerbating the impact on citrus groves^9^.

Traditional methods of disease detection in citrus plants rely heavily on manual inspection by experts, which is time-consuming, labor-intensive, and subject to human error^10^. In very large orchards it is impractical to regularly monitor the many trees, causing delays in disease identification. Additionally, symptoms of different diseases can be visually similar, making accurate diagnosis challenging even for experienced personnel^11^. With the advancements in digital image processing and machine learning technologies, there is a growing interest in developing automated systems for plant disease detection^12,13^. Such systems can deliver rapid, inexpensive and accurate means of monitoring plant health at a large scale. They offer the potential to analyze vast amounts of image data collected through drones, smartphones, or stationary cameras, enabling continuous surveillance of orchards^14–16^.

The application of deep learning in plant disease detection has seen remarkable advancements in recent years, particularly in the classification and diagnosis of crop diseases. Kunduracioglu and Pacal^17^ explored deep learning techniques for grape leaf classification, demonstrating the effectiveness of convolutional neural networks (CNNs) in accurately diagnosing various grape diseases. Their study underscored the potential of deep learning models in enhancing agricultural disease detection, reducing reliance on traditional manual inspections. Similarly, ResNet architectures have been successfully applied for tomato disease identification, as highlighted in Kunduracioglu^18^ research, which focused on leveraging deep residual learning frameworks to improve classification accuracy. The results revealed that deep learning models could achieve superior performance compared to conventional machine learning approaches, reinforcing their viability in precision agriculture.

Furthermore, advancements in disease detection have extended to other crops, including sugarcane and apples. Kunduracıoglu and Paçal^19^ assessed the efficacy of EfficientNet models for sugarcane leaf disease detection, illustrating how optimized deep learning architectures enhance computational efficiency while maintaining high classification accuracy. Their findings suggest that EfficientNet-based approaches provide a promising alternative for large-scale agricultural applications, where processing speed and accuracy are critical factors. Additionally, CNN-based classification methods for apple diseases have been investigated, with Kunduracioglu^20^ proposing robust CNN models capable of distinguishing between various apple pathologies with high precision. This research contributes to the growing body of literature supporting CNNs as a reliable tool for plant disease identification, reinforcing their adaptability across different plant species.

In addition, deep learning, particularly CNNs, has revolutionized the field of computer vision, achieving remarkable success in various image recognition tasks^21,22^. Deep learning models can automatically learn hierarchical feature representations from raw image data, eliminating the need for manual feature extraction^23^. Such a capability transforms them into useful tools for complex pattern recognition tasks such as detection of disease in plant images. Deep learning techniques have been increasingly adopted in the field of the agricultural sector for crop disease detection and classification. For instance, Ergün^24^ explored the use of deep learning methods, specifically DenseNet, to construct robust models capable of accurately distinguishing between different types of citrus anomalies. By studying DenseNet, they were able to confirm that DenseNet is highly accurate in the classification detection of 12 classes of citrus anomalies, reaching 99.50%, which shows deep learning models have strong power in agricultural applications.

Similarly, Omer et al.^25^ proposed a lightweight improved YOLOv5 model for detecting cucumber leaf diseases and pests. In this work, they introduce the convolutional block attention module (CBAM) combined with an optimized architecture to obtain 80.10% mean average precision (mAP) with a model weight size of only 13.6 MB. We highlight the significance of efficient and accurate models for real time agricultural applications, which is the focus of this advancement. In another study, Tejet al.^26^ developed an AI-based smart agriculture 4.0 system for plant disease detection in Tunisia. They used Transfer Learning based ResNet152 Model and various CNN architectures such as ResNet152 and DenseNet121, and achieved an accuracy up to 99%. In their work, they show how advanced deep learning models can strengthen disease identification and management in different agricultural contexts.

Beyond plant diseases, deep learning models have also been applied in medical diagnosis. Jayasudha et al.^27^ introduced a hybrid optimization-enabled deep learning-based ensemble classification for heart disease detection. Through their proposed method, they achieved 94.80% accuracy, proving just one more domain where deep learning can be leveraged across. Furthermore, Türk^28^ investigated machine learning algorithms for heart disease classification using optimization techniques for feature selection. Application of optimization algorithms to ensemble models resulted in improved classification accuracy from 86.34% to 99.08%. The importance of feature selection and model optimization for improving classification performance is demonstrated in this study. In addition, several studies have applied deep learning techniques to detect and classify plant diseases, demonstrating promising results^29,30^. For example, Mohanty et al.^29^ achieved an accuracy of 99.35% in identifying 14 crop species and 26 diseases using a dataset of 54,306 images. Similarly, Ferentinos^30^ reported accuracies exceeding 99% in identifying diseases in five crop species using deep neural networks.

But most of these studies address common crops such as rice, wheat, maize and tomato, generally neglecting citrus plants. Moreover, existing approaches often use small datasets, lack diversity in disease representation, or do not adequately address the challenges posed by varying environmental conditions and disease stages^31^. In the context of citrus disease detection, some studies have explored machine learning methods, but they often suffer from limited sample sizes and constrained scope^32–34^.

The aim of this study is to develop an advanced deep learning-based system to fill these gaps with the detection and classification of citrus leaf and fruit diseases. Tapping on extensive dataset and combining up to date image processing techniques, we aim at improving the accuracy and the robustness of disease identification. In this respect, the proposed methodology consists of several main components.

Firstly, we utilize a comprehensive dataset of citrus disease images obtained from Rauf et al.^35^, including various types of diseases affecting leaves and fruits. We augment our dataset and simulate different disease stages and environmental conditions using advanced techniques to make the model’s generalization capabilities better. Data augmentation strategies such as rotation, scaling, flipping, and color jittering are employed to increase the variability of the training data^36^.

Secondly, we propose sophisticated image enhancement method for preprocessing the images. Techniques such as Contrast Limited Adaptive Histogram Equalization (CLAHE) are used to improve contrast in images with uneven lighting conditions^37^. Advanced segmentation algorithms like U-Net are employed to accurately isolate affected regions, which is critical for focusing the model on relevant features^38^. The segmentation of features helps in improving the performance of detection, specifically reducing the influence of the background noise and irrelevant features.

Thirdly, we employ transfer learning with pre-trained convolutional neural networks to leverage the knowledge embedded in models trained on large-scale image datasets^39^. By fine-tuning models such as EfficientNetB0, ResNet50, InceptionV3, and DenseNet121, we aim to enhance the model’s ability to accurately classify citrus diseases^22,40–42^.

Furthermore, we optimize and validate our models through hyperparameter tuning, regularization techniques, and cross-validation strategies to enhance robustness and generalization^43^. Techniques such as dropout, weight decay, and early stopping are utilized to prevent overfitting and ensure the model performs well on unseen data^44–46^. Additionally, study evaluate the performance of the proposed system using accuracy, precision, recall, F1-score, and Area Under the ROC curve (AUC). We also employ cross-validation strategies and hyperparameter optimization techniques to fine-tune the model and prevent overfitting^43^. The study uses techniques such as *k*-fold cross validation and regularization methods to improve the model and to prove that the model is robust and generalizable. In addition, we incorporate explainable AI methods to interpret the model’s decisions, making the system more transparent and trustworthy for end-users^47,48^. Explainable AI techniques such as Grad-CAM (Gradient-weighted Class Activation Mapping) are used to visualize the regions of the image that the model considers important for making decisions^49^. Such transparency is essential for successful agricultural professionals to trust the model and to be able to spot errors in its predictions.

In brief, this research aims to contribute to the field of agricultural image analysis through offering a simple and usable tool for citrus disease management. Early disease detection is seen to assist farmers and agricultural professionals, to intervene effectively and thus reducing crop losses. We combine advanced deep learning models with practical implementation strategies in order to bridge the gap from research to real world.

The rest of this paper is organized from here onwards. Section “Materials and methods” describes the materials and methods used, including dataset preparation, image preprocessing, and model development. Section “Results and discussion” presents the results of the experiments and discusses the findings and their implications. Finally, Section “Conclusion” concludes the paper and suggests directions for future research.

## Materials and methods

### Dataset collection and annotation

The dataset utilized in this research was obtained from Rauf et al.^35^, providing a comprehensive collection of 759 images for the detection and classification of citrus diseases using machine learning techniques. The images encompass both healthy and diseased citrus fruits and leaves, targeting diseases such as Black spot, Canker, Scab, Greening, and Melanose, which are significant threats to citrus production worldwide^3,4^. The image size is $256 \times 256$ pixels at a resolution of 72 dots per inch (dpi), each image captured under variable conditions that emulate real world scenarios such as changes of lighting, background and outside elements. This diversity enhances the robustness of the dataset, allowing for the development of models that generalize well to new, unseen data^50,51^.

The dataset is organized into two main categories: citrus leaves and citrus fruits. For citrus fruits, there are 150 images distributed among five classes: Other diseases are Black spot, Canker, Greening, Scab and Healthy. For citrus leaves, there are 609 images categorized into five classes: Greening, Black spot, Healthy, Canker, and Melanose. The distribution of images across these classes is detailed in Table 1.

**Table 1:** Distribution of images in the citrus dataset.

Class	Citrus fruits	Citrus leaves
Black spot	19	171
Canker	78	163
Greening	16	204
Healthy	22	58
Melanose	–	13
Scab	15	–
Total	150	609

High quality labels for the dataset were carefully annotated by plant pathology experts. We took each image and laboriously classified the image based on the visual symptoms that correspond to each disease. The use of expert annotations enhances the reliability of the dataset and is crucial for training accurate machine learning models^29^. To address potential class imbalances, particularly the fewer number of healthy leaf images compared to diseased ones, strategies such as oversampling the minority class and using class weights during training were considered^22^. Since we have to prevent the model from biased due to majority classes, we need to balance the dataset.

The dataset was partitioned into training, validation, and testing sets using a stratified sampling approach to maintain the proportional representation of each class in all subsets^52^. Particularly, 70 % of data was used for training, 15 % for validation and 15 % for testing. For Deep Learning models, the training set was used to fit, the validation set to tune Hyperparameters and avoid overfitting and the test set to report the final model’s performance.

### Data augmentation and preprocessing

Data augmentation and preprocessing steps were implemented to improve the model robustness and generalization capabilities. Given the limited size of the dataset, data augmentation was essential to prevent overfitting and improve model performance on unseen data^36^. The original images were transformed as a series of transformations applied, to create a more diverse training set. The augmentation techniques applied were:

- **Geometric transformations**: Random rotations within a range of $- \,30^\circ$ to $+\,30^\circ$, horizontal and vertical flipping, and scaling between 0.8 to 1.2 times the original size. These transformations help the model become invariant to orientation and scale variations^21^.
- **Translation and shearing**: Random translations up to 10% of the image dimensions and shearing transformations to simulate changes in viewpoint and introduce variability in the spatial arrangement of disease symptoms.
- **Color space adjustments**: Modifications to brightness, contrast, saturation, and hue to mimic different lighting conditions encountered in real-world scenarios^53^.
- **Noise injection**: Addition of Gaussian noise to a subset of images to improve the model’s resilience to image noise resulting from sensor imperfections or compression artifacts.

**Fig. 1:**
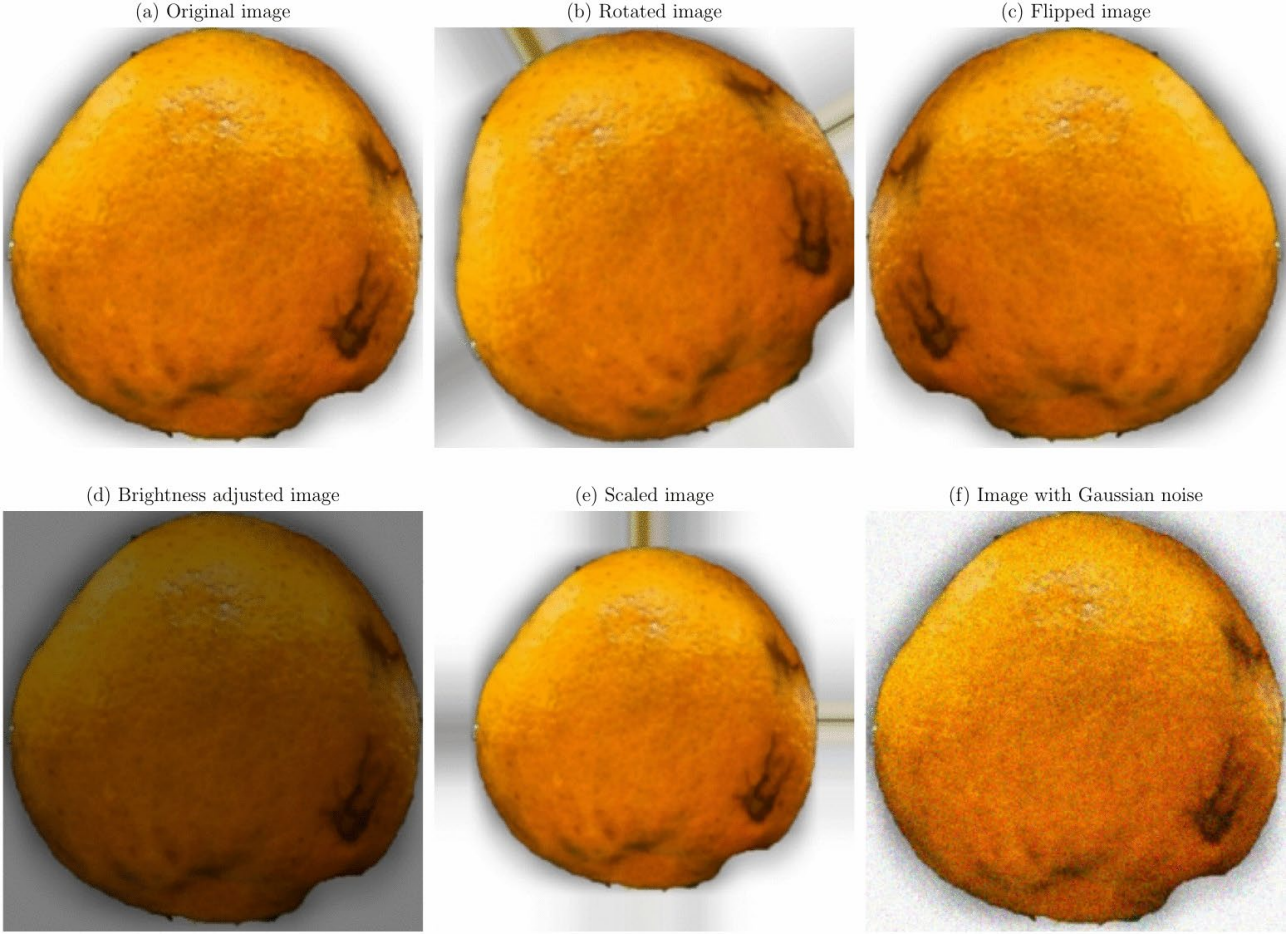
Data augmentation applied to a citrus fruit image affected by Black spot disease: (**a**) Original image, (**b**) Rotated image, (**c**) Flipped image, (**d**) Brightness adjusted image, (**e**) Scaled image, (**f**) Image with Gaussian noise.

**Fig. 2:**
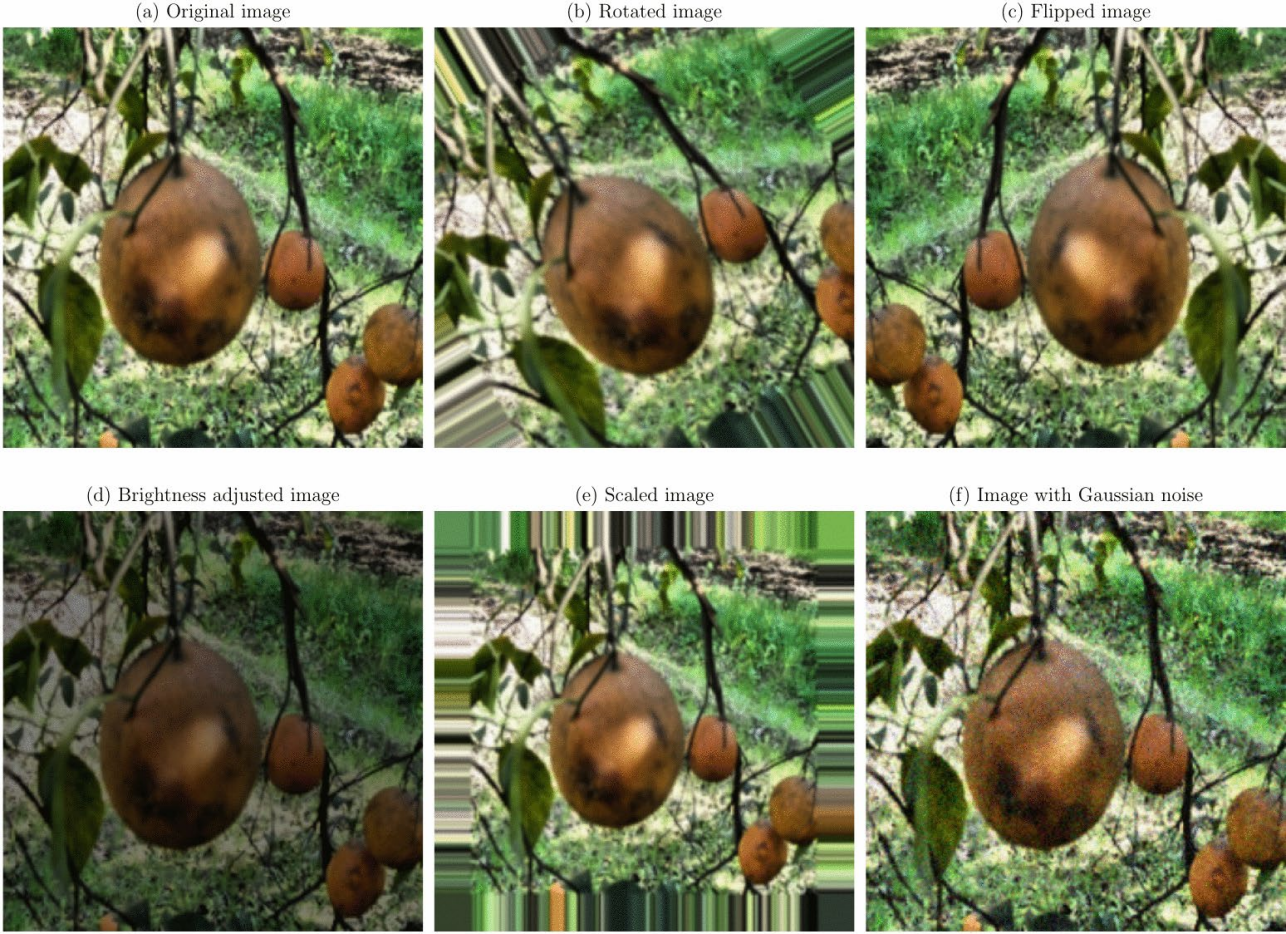
Data augmentation applied to a citrus fruit image affected by Canker disease: (**a**) Original image, (**b**) Rotated image, (**c**) Flipped image, (**d**) Brightness adjusted image, (**e**) Scaled image, (**f**) Image with Gaussian noise.

**Fig. 3:**
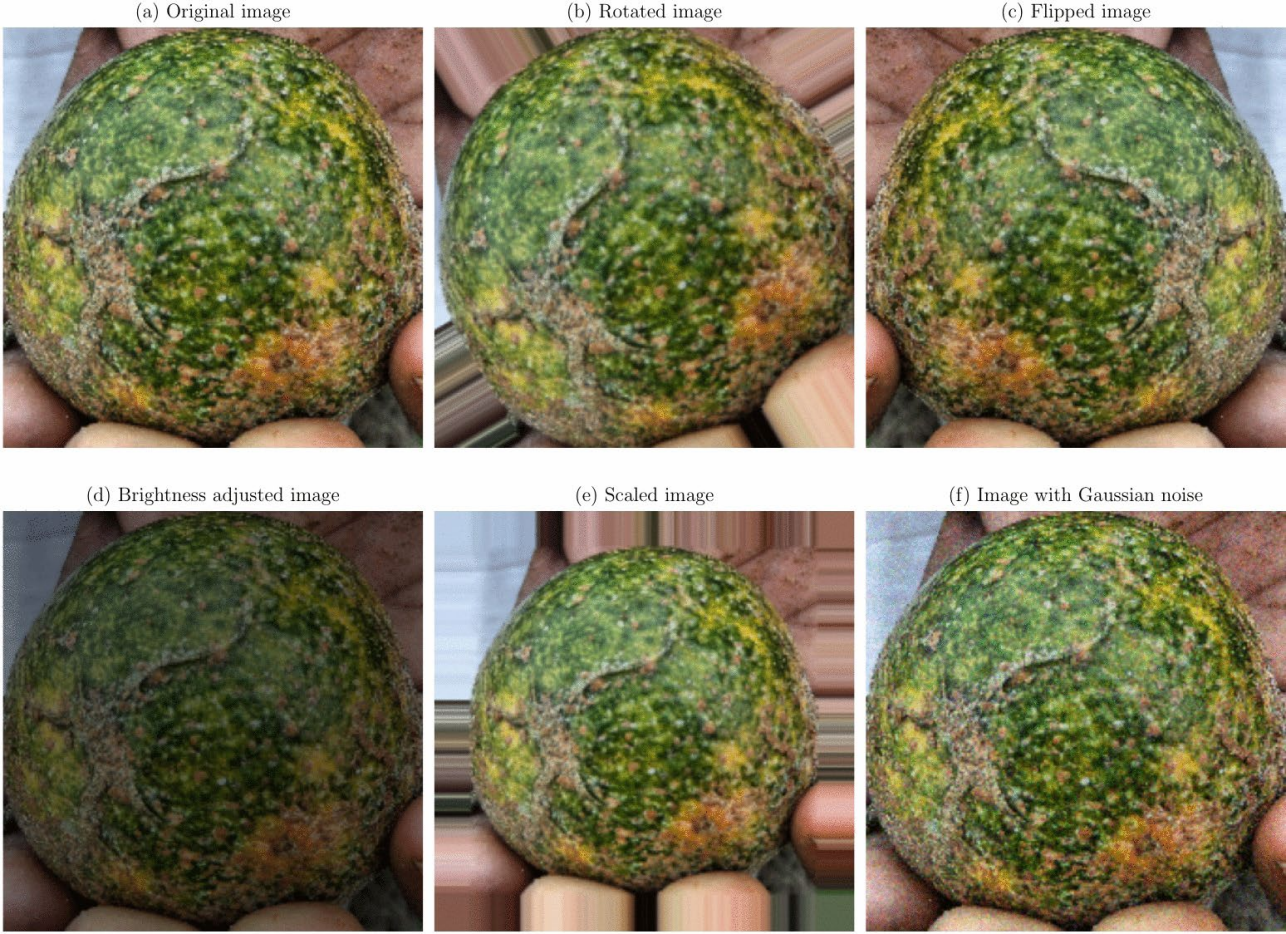
Data augmentation applied to a citrus fruit image affected by Greening disease: (**a**) Original image, (**b**) Rotated image, (**c**) Flipped image, (**d**) Brightness adjusted image, (**e**) Scaled image, (**f**) Image with Gaussian noise.

**Fig. 4:**
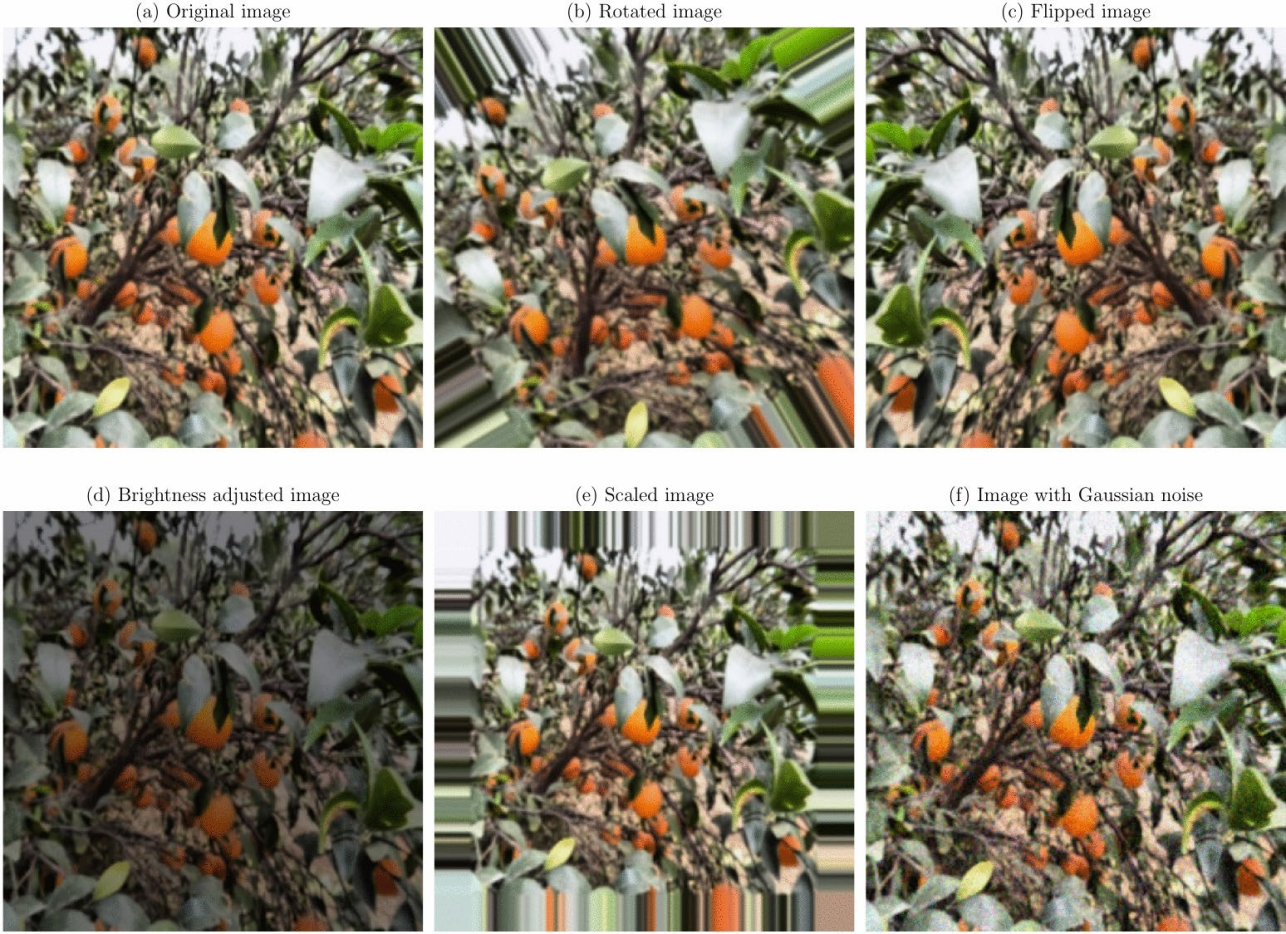
Data augmentation applied to a healthy citrus fruit image: (**a**) Original image, (**b**) Rotated image, (**c**) Flipped image, (**d**) Brightness adjusted image, (**e**) Scaled image, (**f**) Image with Gaussian noise.

**Fig. 5:**
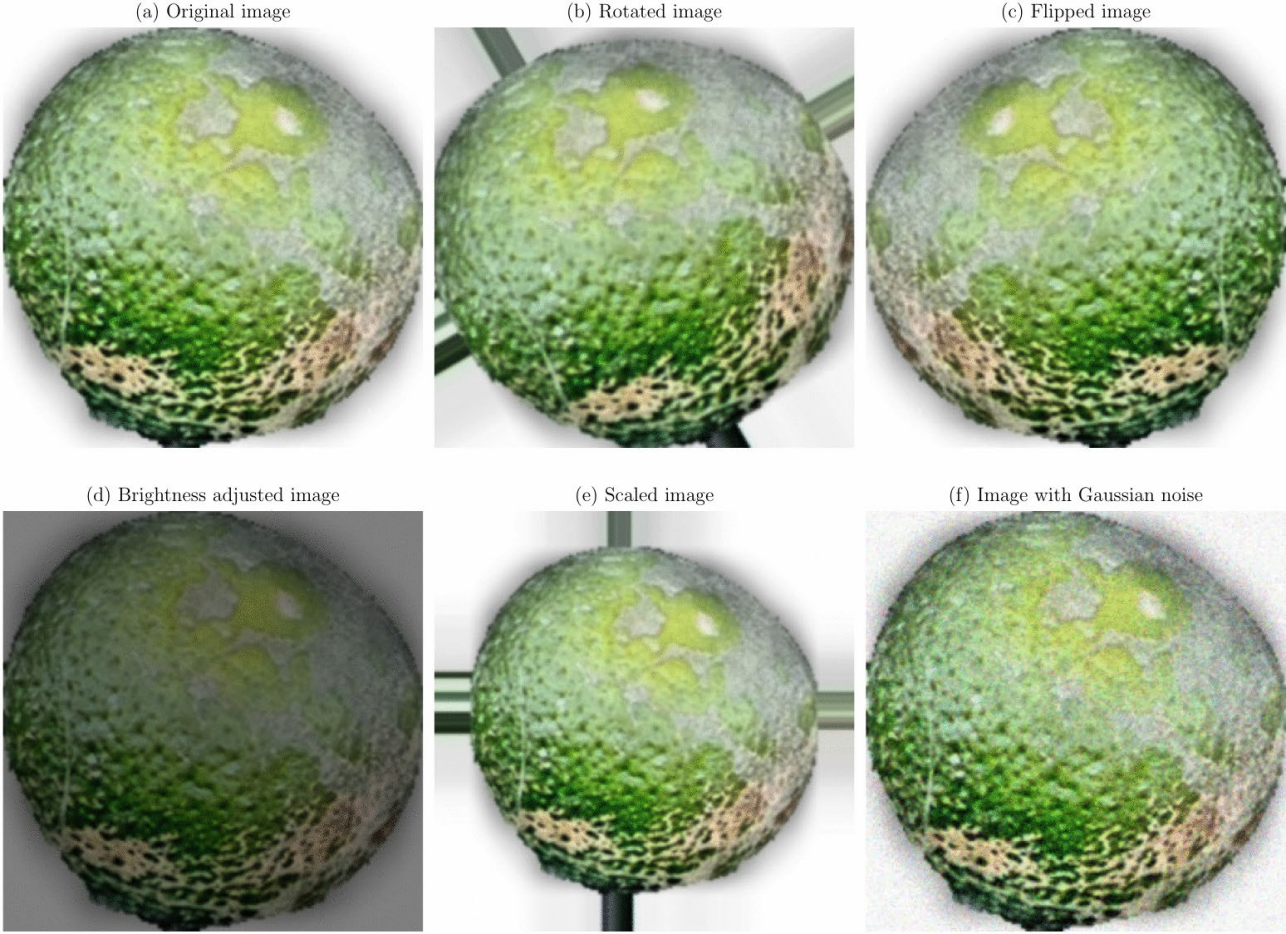
Data augmentation applied to a citrus fruit image affected by Scab disease: (**a**) Original image, (**b**) Rotated image, (**c**) Flipped image, (**d**) Brightness adjusted image, (**e**) Scaled image, (**f**) Image with Gaussian noise.

**Fig. 6:**
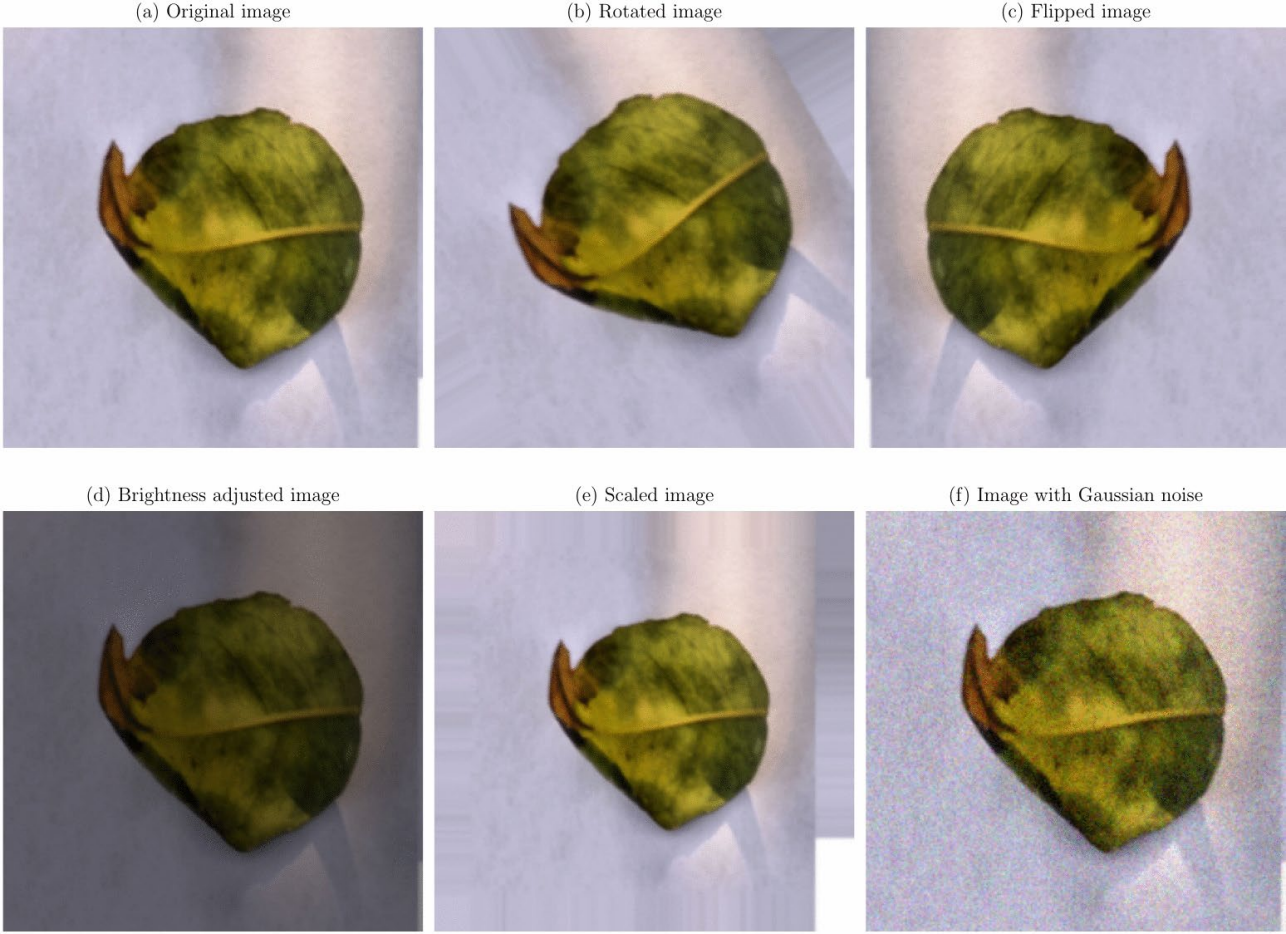
Data augmentation applied to a citrus leaf image affected by Black spot disease: (**a**) Original image, (**b**) Rotated image, (**c**) Flipped image, (**d**) Brightness adjusted image, (**e**) Scaled image, (**f**) Image with Gaussian noise.

**Fig. 7:**
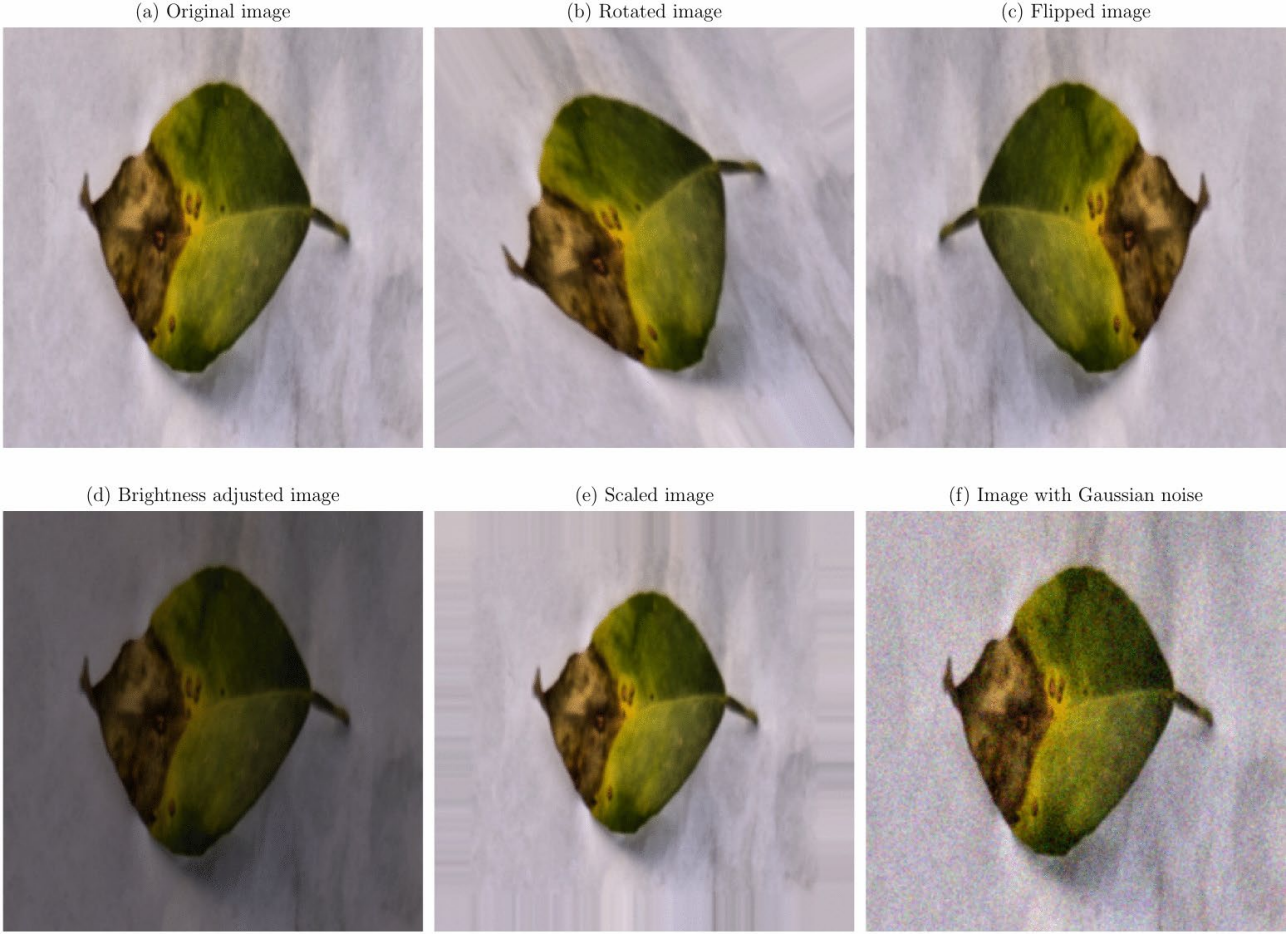
Data augmentation applied to a citrus leaf image affected by Canker disease: (**a**) Original image, (**b**) Rotated image, (**c**) Flipped image, (**d**) Brightness adjusted image, (**e**) Scaled image, (**f**) Image with Gaussian noise.

**Fig. 8:**
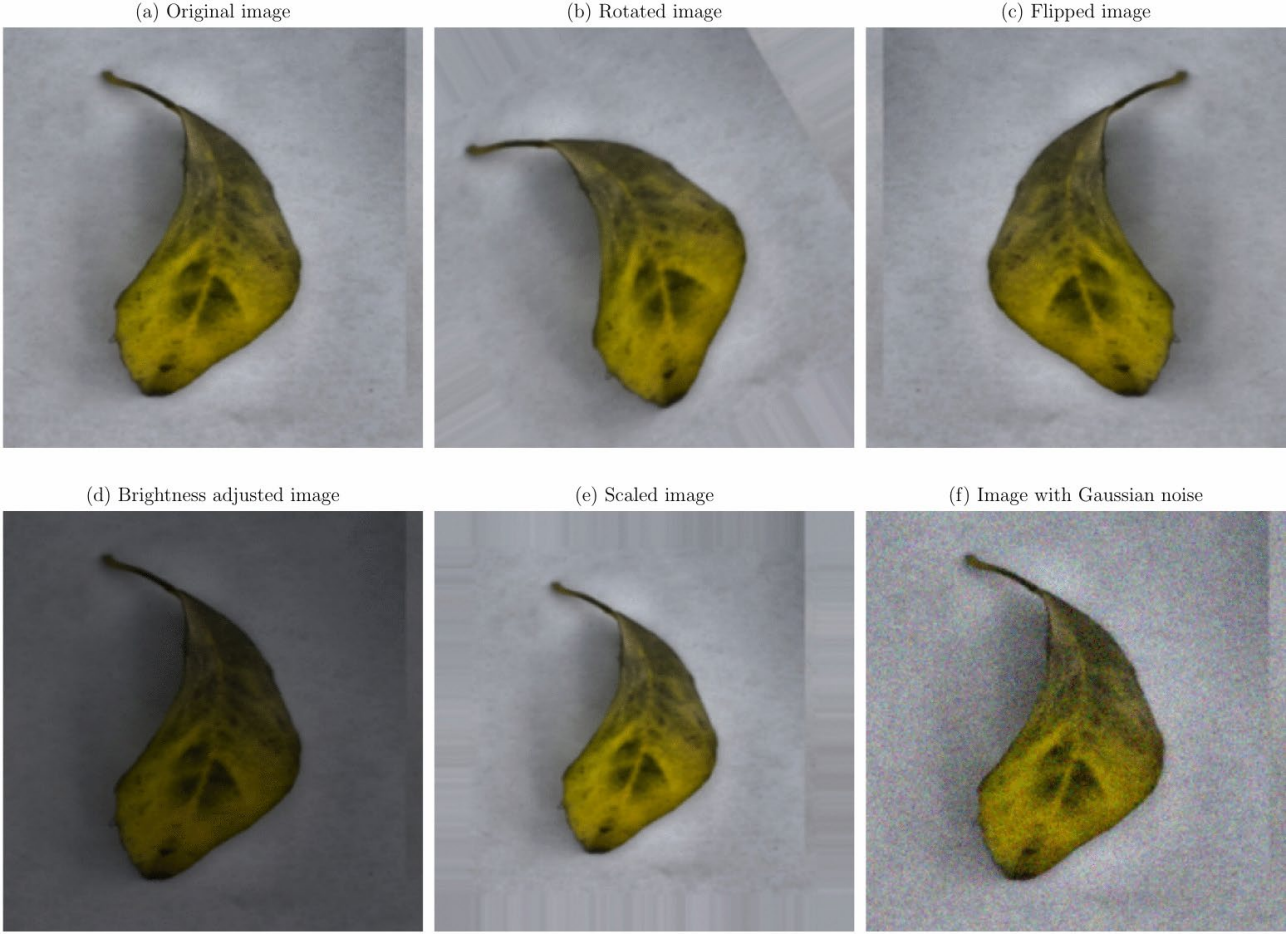
Data augmentation applied to a citrus leaf image affected by Greening disease: (**a**) Original image, (**b**) Rotated image, (**c**) Flipped image, (**d**) Brightness adjusted image, (**e**) Scaled image, (**f**) Image with Gaussian noise.

**Fig. 9:**
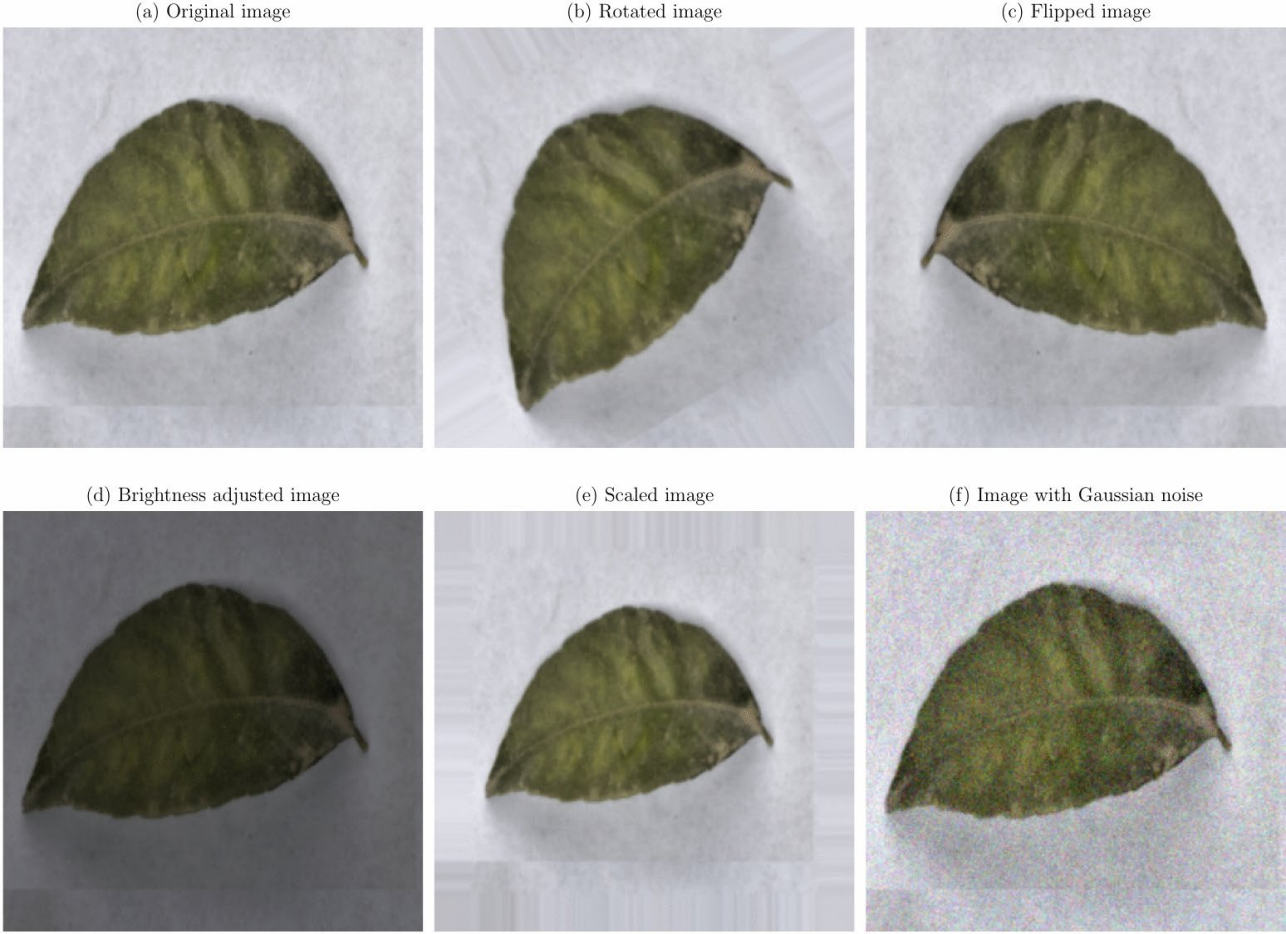
Data augmentation applied to a healthy citrus leaf image: (**a**) Original image, (**b**) Rotated image, (**c**) Flipped image, (**d**) Brightness adjusted image, (**e**) Scaled image, (**f**) Image with Gaussian noise.

**Fig. 10:**
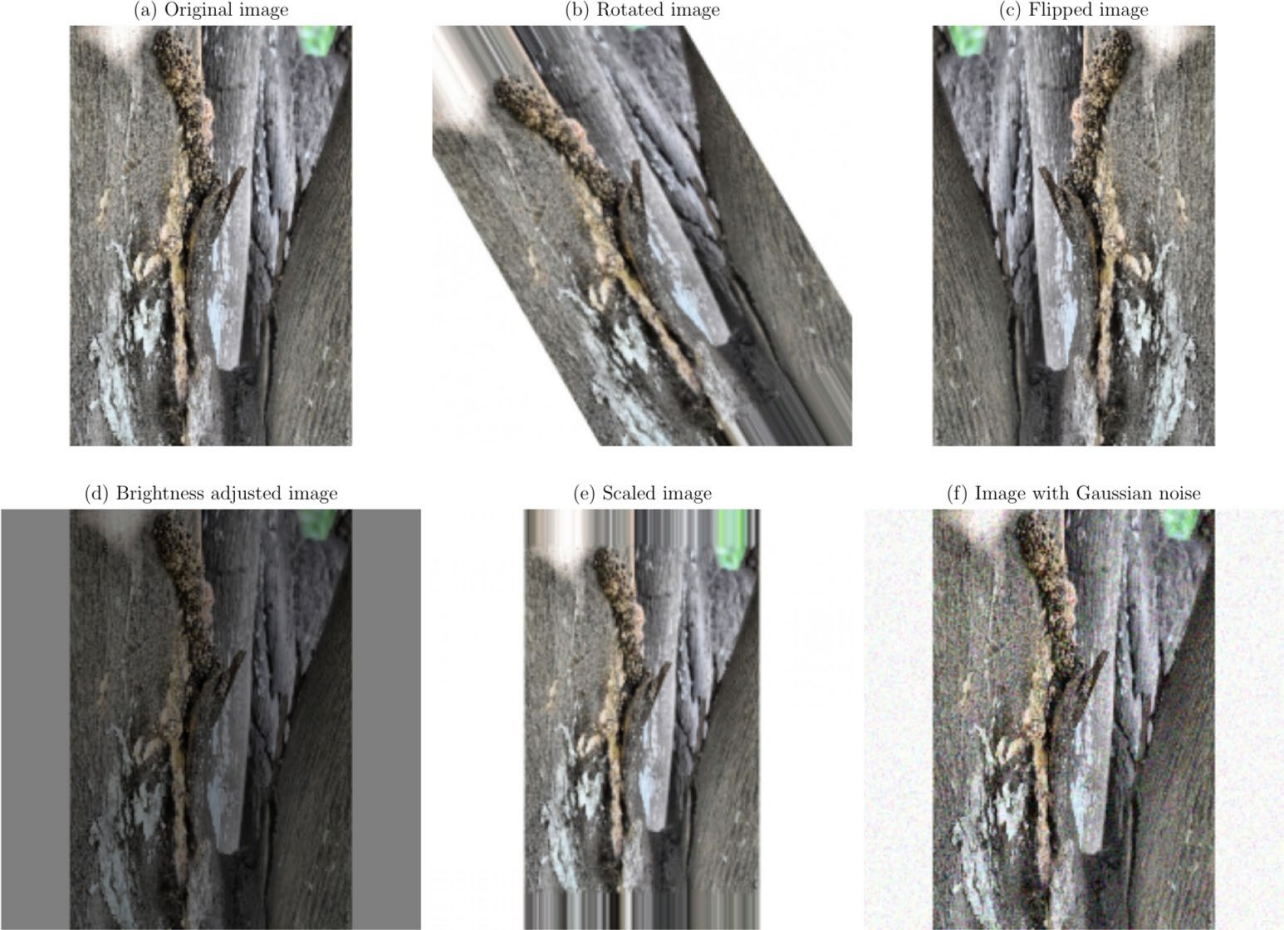
Data augmentation applied to a citrus leaf image affected by Melanose disease: (**a**) Original image, (**b**) Rotated image, (**c**) Flipped image, (**d**) Brightness adjusted image, (**e**) Scaled image, (**f**) Image with Gaussian noise.

Figures 1, 2, 3, 4, 5, 6, 7, 8, 9 and 10 illustrate the data augmentation techniques applied to sample images from each class in both citrus fruits and leaves categories. The original image (a) is rotated (b), flipped (c), adjusted for brightness (d), scaled (e), and the Gaussian noise is added (f) for each figure. The training data is augmented with these images, and is diverse, such that the models can learn robust features that generalize well to new images. The next series of figures shows data augmentation applied to each class within each category, citrus fruits and leaves. For each figure, I have the original image and representations of the augmented versions, demonstrating multiple transformations that were used to create the augmented training dataset.

For preprocessing, all images were resized to $224 \times 224$ pixels to match the input size required by the deep learning models^22,41^. Pixel values were normalized to the range [0, 1], and mean subtraction was performed using the ImageNet dataset’s mean values to standardize the input data^39^.

### Image enhancement techniques

Image enhancement techniques were applied to improve the quality of images for better analysis and interpretation. Enhancing the visual features of diseased regions aids deep learning models in accurately identifying and classifying diseases^54^. The primary methods employed included:

- **Contrast enhancement using CLAHE**: Contrast Limited Adaptive Histogram Equalization (CLAHE) was utilized to enhance the local contrast of images, improving the visibility of features in both bright and dark regions^37^. The CLAHE algorithm modifies the pixel intensity values based on their local neighborhood, preventing over-amplification of noise.
- **Noise reduction with median filtering**: Median filtering was applied to reduce impulsive noise while preserving edges and fine details critical for disease detection^55^. The median filter replaces each pixel’s value with the median of neighboring pixel values within a defined window, effectively removing outliers.
- **Color space transformation**: Images were transformed from the RGB color space to the HSV (Hue, Saturation, Value) color space to decouple color information from intensity, allowing for more effective processing of color features^56^.

**Fig. 11:**
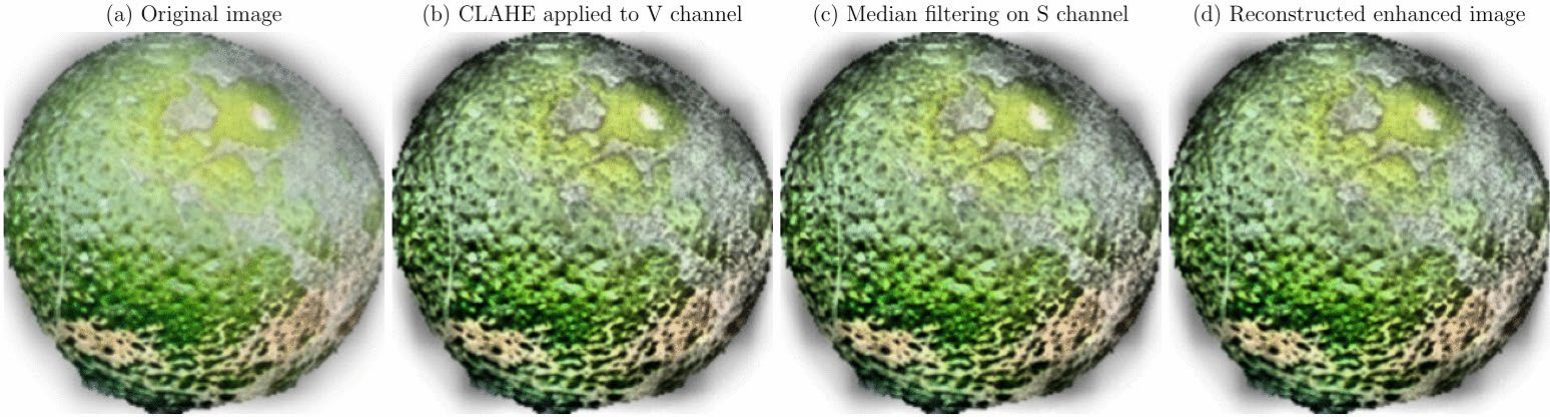
Image enhancement pipeline for citrus fruits: (**a**) Original image, (**b**) CLAHE applied to V channel, (**c**) Median filtering on S channel, (**d**) Reconstructed enhanced image.

**Fig. 12:**
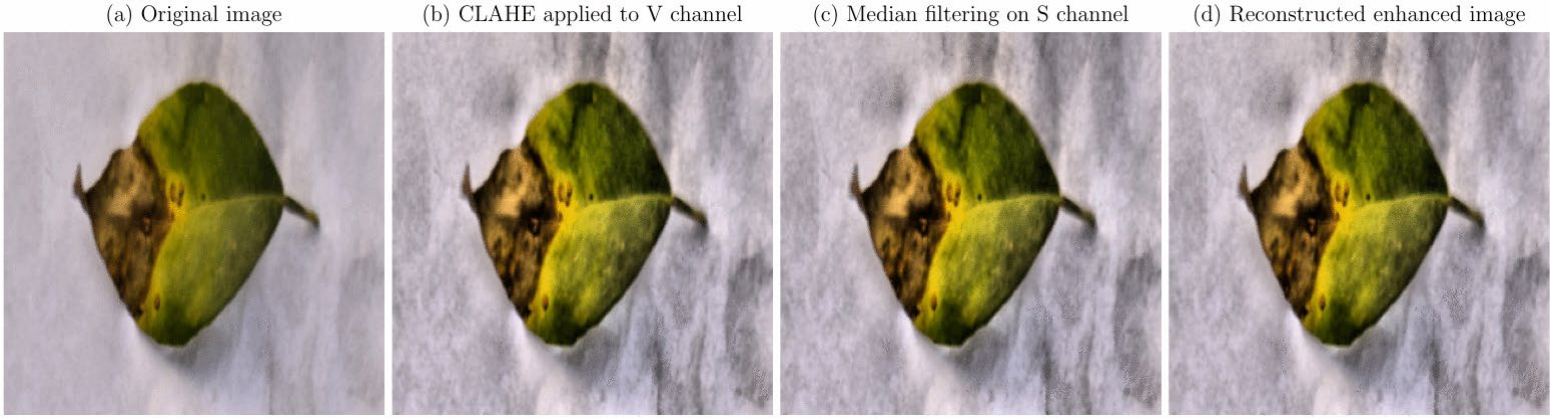
Image enhancement pipeline for citrus leaves: (**a**) Original image, (**b**) CLAHE applied to V channel, (**c**) Median filtering on S channel, (**d**) Reconstructed enhanced image.

These techniques were integrated into a preprocessing pipeline applied to all images before model training. The steps included conversion to HSV color space, application of CLAHE to the V channel, median filtering on the S channel, and reconstruction of the enhanced image. The enhancement processes for citrus fruits and leaves are illustrated in Figs. 11 and 12, respectively. Figure 11 shows the enhancement pipeline for citrus fruits, while Fig. 12 illustrates the enhancement pipeline for citrus leaves. The original images are displayed in (a), followed by the CLAHE-enhanced V channel in (b), median-filtered S channel in (c), and the reconstructed enhanced image in (d).

### Feature extraction methods

Feature extraction was performed using CNNs, which automatically learn hierarchical feature representations from raw image data^23^. Several pre-trained CNN architectures were considered, including VGG16^57^, ResNet50^22^, InceptionV3^41^, and EfficientNetB0^40^.

Transfer learning was employed to leverage the knowledge embedded in pre-trained models trained on the ImageNet dataset^39^. The pre-trained models were modified by replacing the final classification layers with new fully connected layers tailored to the number of classes in the citrus disease dataset. The general architecture modifications included:

- **Global average pooling layer**: To reduce the spatial dimensions of the feature maps while retaining important information.
- **Fully connected dense layers**: One or more dense layers with ReLU activation functions to learn non-linear combinations of the high-level features.
- **Dropout layers**: Applied with a dropout rate of 0.5 to prevent overfitting by randomly deactivating neurons during training^44^.
- **Output layer**: A softmax activation function was used in the final layer to output probability distributions over the classes.

### Classification models

Several deep learning classification models were developed, utilizing the features extracted by the CNNs. The primary models included fine-tuned versions of EfficientNetB0, ResNet50, InceptionV3, and DenseNet121^22,40–42^.

The training procedure involved two phases:

1. **Training the top layers**: The convolutional base was frozen, and only the added top layers were trained using the citrus disease dataset. The Adam optimizer with a learning rate of $1 \times 10^{-3}$ and categorical cross-entropy as the loss function was used^58^.
2. **Fine-tuning the convolutional base**: Selected layers from the convolutional base were unfrozen and fine-tuned alongside the top layers with a lower learning rate of $1 \times 10^{-5}$^59^.

### Model optimization and validation

Model optimization and validation were conducted to develop robust and generalizable deep learning models. Several hyperparameters were selected for optimization, including learning rate, batch size, number of epochs, optimizer type, and the number of layers to fine-tune^43^. The ranges and selected values for these hyperparameters are detailed in Table 2.

**Table 2:** Hyperparameters selected for optimization with their respective ranges.

Hyperparameter	Range	Selected value
Learning rate	$[1 \times 10^{-5}, 1 \times 10^{-2}]$	$1 \times 10^{-3}$
Batch size	$\{16, 32, 64\}$	32
Number of epochs	[10, 100]	50
Optimizer type	{SGD, Adam, RMSprop}	Adam
Number of layers to fine-tune	[5, 20] layers	20 layers

Regularization techniques were employed to prevent overfitting and improve generalization:

- **Dropout**: Applied dropout layers with a rate of 0.5 after fully connected layers, randomly deactivating neurons during training to prevent co-adaptation of features^44^.
- **Weight Decay (L2 Regularization)**: Added an L2 penalty to the loss function to discourage large weights^45^. The total loss function with L2 regularization is calculated using Eq. (1).
  1 $$\begin{aligned} \mathcal {L}_{\text {total}} = \mathcal {L}_{\text {data}} + \lambda \sum _{i} w_{i}^{2} \end{aligned}$$
  where $\mathcal {L}_{\text {data}}$ is the original loss function (categorical cross-entropy), $\lambda$ is the regularization parameter controlling the strength of the penalty, and $w_{i}$ are the weights of the model.
- **Early stopping**: Monitored the validation loss during training and implemented early stopping if the loss did not improve for a set number of epochs (patience), preventing overfitting by halting training before the model begins to memorize the training data^46^.

The dataset was split into training, validation, and test sets using a stratified sampling approach to maintain the proportional representation of each class^52^. Additionally, *k*-fold cross-validation with $k=5$ was employed to ensure that each sample was used for validation exactly once, enhancing the reliability of the model evaluation^60^. Model performance was assessed using evaluation metrics including accuracy, precision, recall, F1-score, and the ROC and AUC^61^.

Accuracy measures the overall correctness of the model and is given by Eq. (2).

2 $$\begin{aligned} \text {Accuracy} = \frac{\text {TP} + \text {TN}}{\text {TP} + \text {FP} + \text {TN} + \text {FN}} \end{aligned}$$

where $\text {TP}$ is the number of true positives, $\text {TN}$ is true negatives, $\text {FP}$ is false positives, and $\text {FN}$ is false negatives.

Precision quantifies the correctness of positive predictions and is given by Eq. (3).

3 $$\begin{aligned} \text {Precision} = \frac{\text {TP}}{\text {TP} + \text {FP}} \end{aligned}$$

Recall, also known as sensitivity or true positive rate, measures the model’s ability to identify positive instances, given by Eq. (4).

4 $$\begin{aligned} \text {Recall} = \frac{\text {TP}}{\text {TP} + \text {FN}} \end{aligned}$$

The F1-score provides a harmonic mean of precision and recall, given by Eq. (5).

5 $$\begin{aligned} \text {F1-Score} = 2 \times \frac{\text {Precision} \times \text {Recall}}{\text {Precision} + \text {Recall}} \end{aligned}$$

The ROC curve is plotted by varying the classification threshold and plotting the True Positive Rate (TPR) against the False Positive Rate (FPR). The AUC provides a single measure of overall model performance, representing the likelihood that the model ranks a random positive instance higher than a random negative instance^61^. By optimizing the hyperparameters as shown in Table 2 and employing these evaluation metrics, the models were fine-tuned to achieve high accuracy and generalization capability on unseen data.

### Explainability and model interpretability

To enhance trust in deep learning models and facilitate their adoption in agricultural settings, we integrated Gradient-weighted Class Activation Mapping (Grad-CAM) as an interpretability tool. Grad-CAM generates heatmaps that highlight the most influential regions in an image that contributed to the model’s classification decision^49^.

We applied Grad-CAM to both correctly classified and misclassified samples, allowing us to verify whether the model focused on disease-affected regions or was influenced by background noise. The Grad-CAM heatmaps provide visual evidence of feature importance, helping agricultural professionals understand model predictions and gain confidence in its recommendations. Additionally, the generated heatmaps assist in identifying potential false positives and false negatives, enabling further refinement of the model for practical deployment. The integration of explainable AI methods enhances the transparency and interpretability of the deep learning models, making them more accessible and trustworthy for end-users^47,48^.

## Results and discussion

The performance of four deep learning models, EfficientNetB0, ResNet50, InceptionV3, and DenseNet121, was thoroughly evaluated to determine their efficacy in classifying citrus diseases. This section presents the performance metrics, conducts a comparative analysis of the models, discusses the implications for citrus disease management, and outlines the limitations and potential avenues for future research.

### Comparison models’ performance

The test performance of the four models is summarized in Table 3. Both InceptionV3 and DenseNet121 achieved the highest test accuracy of 99.12%, with very low test losses of 0.02496 and 0.02142, respectively, demonstrating a remarkable improvement after hyperparameter tuning and model optimization. ResNet50 and EfficientNetB0 attained test accuracies of 84.58% and 80.18%, with test losses of 0.36849 and 0.49724, respectively. These outcomes underscore the superior capability of InceptionV3 and DenseNet121 in accurately classifying citrus diseases within the dataset.

**Table 3:** Test loss and accuracy of classification models.

Model	Test loss	Test accuracy (%)
EfficientNetB0	0.49724	80.18
ResNet50	0.36849	84.58
InceptionV3	0.02496	99.12
DenseNet121	0.02142	99.12

Furthermore, the impact of hyperparameter tuning and advanced data augmentation on model performance was substantial. Table 4 presents the validation loss and accuracy of each model after applying these enhancements. The results indicate that InceptionV3 and DenseNet121 achieved the highest validation accuracy of 99.12% with RMSprop (learning rate = 0.001), whereas ResNet50 and EfficientNetB0 attained validation accuracies of 80.62% and 80.18%, respectively. These improvements validate the effectiveness of optimizing learning rates and incorporating Mixup augmentation in deep learning models for citrus disease classification.

**Table 4:** Optimized hyperparameters and model performance.

Model	Best optimizer	Best LR	Validation loss	Validation accuracy
EfficientNetB0	Adam	0.001	0.4974	80.18%
ResNet50	Adam	0.001	0.4158	80.62%
InceptionV3	RMSprop	0.001	0.0305	99.12%
DenseNet121	RMSprop	0.001	0.0262	99.12%

The results show that hyperparameter optimization significantly enhanced model accuracy, particularly for InceptionV3 and DenseNet121, which both achieved near-perfect classification performance. ResNet50 and EfficientNetB0, while showing moderate improvements, benefited from Mixup augmentation, allowing for better generalization in complex classification tasks.

### Comparative analysis

In this section, we present a comparative analysis of four deep learning models-EfficientNetB0, ResNet50, InceptionV3, and DenseNet121-by evaluating their performance on citrus disease classification under the tuned hyperparameters. The classification reports, confusion matrices, training curves, and ROC curves are based on these optimized settings, and the results demonstrate significant improvements in accuracy, precision, recall, and F1-score, especially for InceptionV3 and DenseNet121. This improvement underscores the importance of meticulous hyperparameter tuning and model optimization in achieving state-of-the-art performance.

Table 5 provides the classification reports for each model, separating the performance for Fruits and Leaves, along with the macro and weighted averages. As shown, DenseNet121 and InceptionV3 both achieve an overall accuracy of approximately 99.12%, with near-perfect precision, recall, and F1-scores for both Fruits and Leaves. ResNet50 and EfficientNetB0 also exhibit better results than previously reported, with ResNet50 achieving an accuracy of 84.58% and EfficientNetB0 improving to 80.18%. However, DenseNet121 and InceptionV3 still maintain a clear advantage in terms of balanced metrics across both Fruits and Leaves classes. These findings indicate that deeper or more sophisticated architectures with properly fine-tuned parameters can substantially enhance classification outcomes, which aligns well with earlier research that has consistently highlighted the benefits of deeper CNN models in complex image recognition tasks.

**Table 5:** Classification reports of deep learning models.

Model and class	Precision	Recall	F1-Score	Support
DenseNet121 (Fruits)	0.9574	1.0000	0.9783	45
DenseNet121 (Leaves)	1.0000	0.9890	0.9945	182
DenseNet121 (accuracy)	0.9912			
DenseNet121 (macro avg)	0.9787	0.9945	0.9864	227
DenseNet121 (weighted avg)	0.9916	0.9912	0.9913	227
EfficientNetB0 (Fruits)	0.0000	0.0000	0.0000	45
EfficientNetB0 (Leaves)	0.8018	1.0000	0.8900	182
EfficientNetB0 (accuracy)	0.8018			
EfficientNetB0 (macro avg)	0.4009	0.5000	0.4450	227
EfficientNetB0 (weighted avg)	0.6428	0.8018	0.7135	227
ResNet50 (Fruits)	0.9167	0.2444	0.3860	45
ResNet50 (Leaves)	0.8419	0.9945	0.9118	182
ResNet50 (accuracy)	0.8458			
ResNet50 (macro avg)	0.8793	0.6195	0.6489	227
ResNet50 (weighted avg)	0.8567	0.8458	0.8076	227
InceptionV3 (Fruits)	0.9778	0.9778	0.9778	45
InceptionV3 (Leaves)	0.9945	0.9945	0.9945	182
InceptionV3 (accuracy)	0.9912			
InceptionV3 (macro avg)	0.9861	0.9861	0.9861	227
InceptionV3 (weighted avg)	0.9912	0.9912	0.9912	227

In light of these metrics, it is evident that DenseNet121 and InceptionV3 stand out for their ability to consistently detect both Fruits and Leaves diseases with exceptionally high precision and recall. The minimal difference between their macro and weighted averages also demonstrates that they can manage potential class imbalance effectively, which is crucial in real-world scenarios where certain diseases or classes may be underrepresented. ResNet50 exhibits moderate accuracy, primarily due to better performance in classifying Leaves compared to Fruits. Nevertheless, its performance lags behind the top two models, suggesting that deeper fine-tuning or additional data augmentation might further improve its results. EfficientNetB0, despite a notable improvement to an accuracy of 80.18%, still struggles significantly with the Fruits class, as reflected by zero precision and recall. This disparity suggests that certain architectural characteristics or hyperparameter settings might be less suited for the subtle distinctions required to classify diseased citrus fruits accurately.

**Fig. 13:**
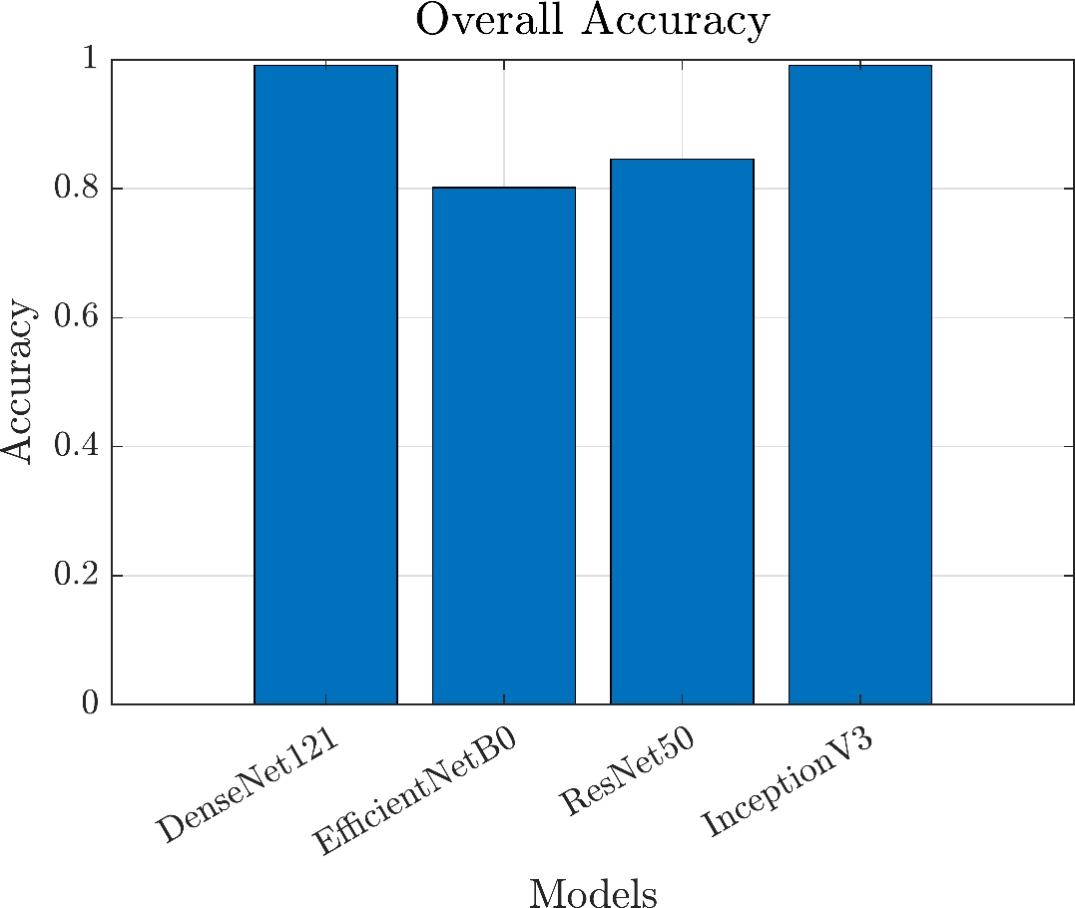
Overall accuracy comparison across the four models.

**Fig. 14:**
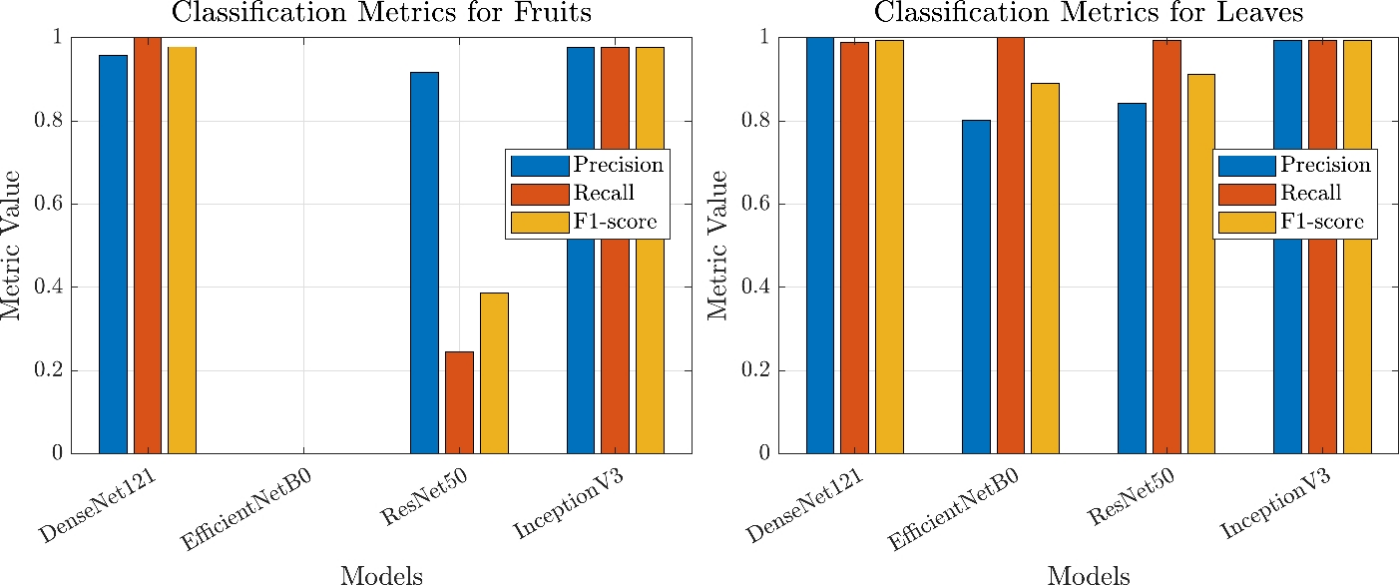
Precision, Recall, and F1-scores for Fruits (left) and Leaves (right) across the Four Models.

Figures 13, and 14 show a comparison of overall accuracy as well as precision, recall, and F1-scores for both Fruits and Leaves. In these plots, DenseNet121 and InceptionV3 consistently hover around near-perfect scores, whereas ResNet50 and EfficientNetB0 display more variability. These findings are consistent with earlier studies that emphasize the value of deeper convolutional architectures and the necessity of careful parameter tuning for image-based plant disease classification. The high accuracy reported here aligns with prior work on transfer learning approaches in agricultural domains, thereby reinforcing the notion that robust deep learning models can offer significant advantages in real-world applications such as disease monitoring and precision agriculture.

**Fig. 15:**
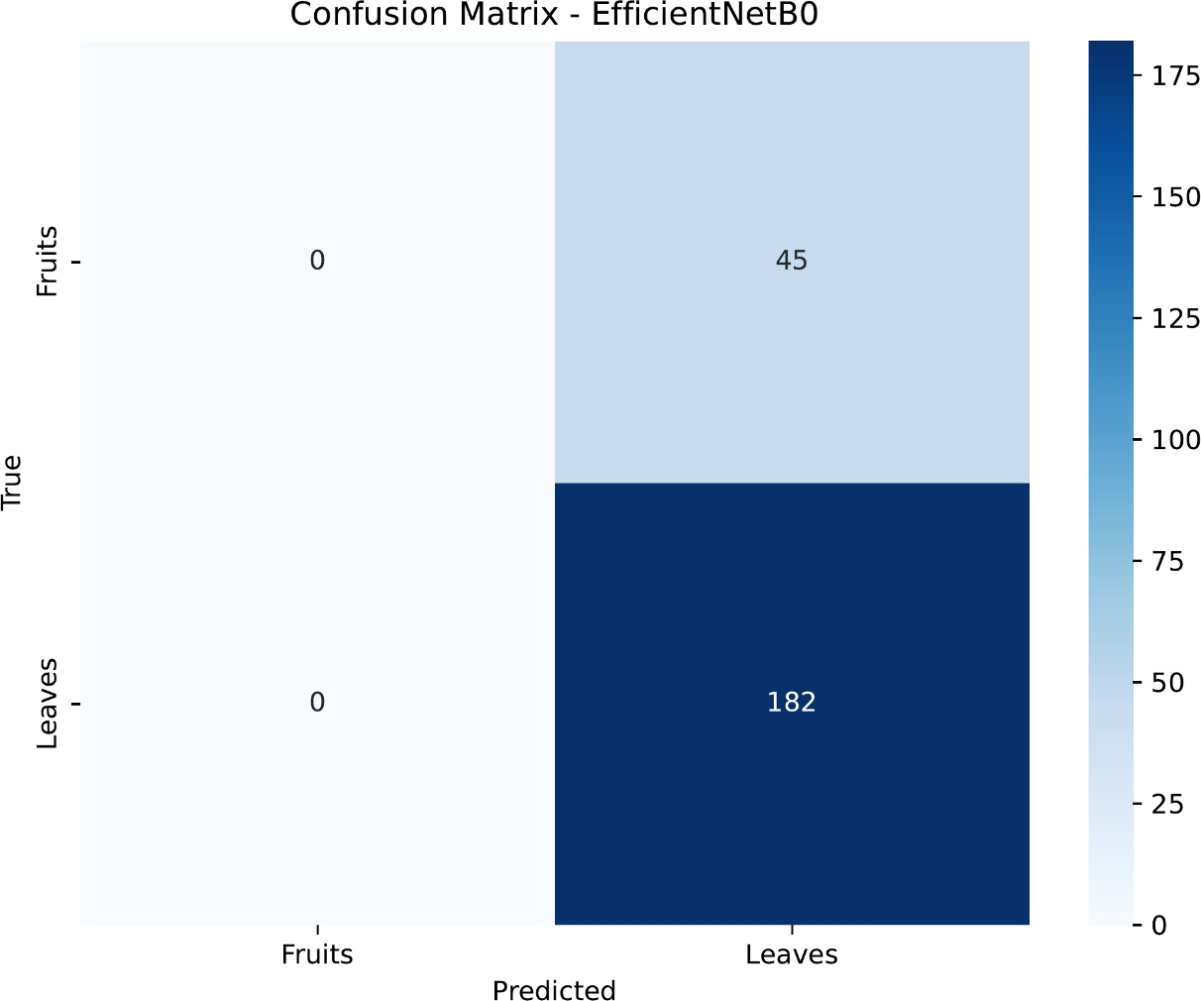
Confusion matrix for the EfficientNetB0 model.

**Fig. 16:**
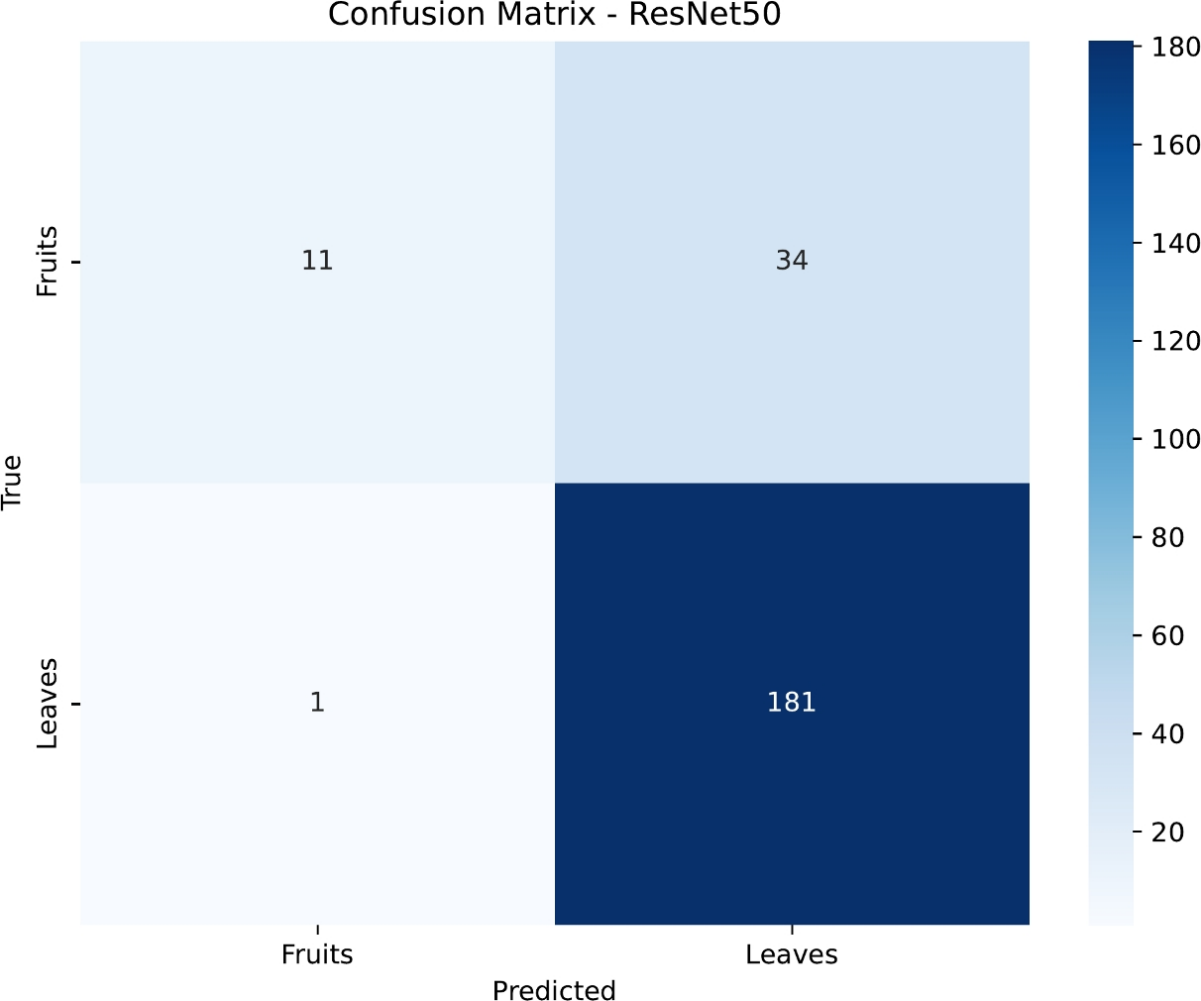
Confusion matrix for the ResNet50 model.

**Fig. 17:**
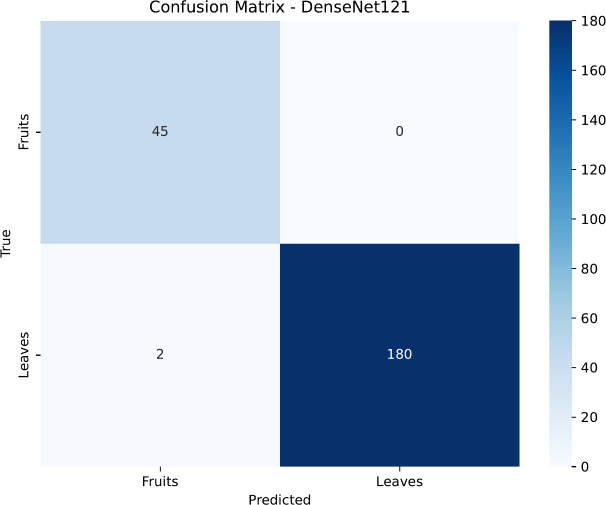
Confusion matrix for the DenseNet121 model.

**Fig. 18:**
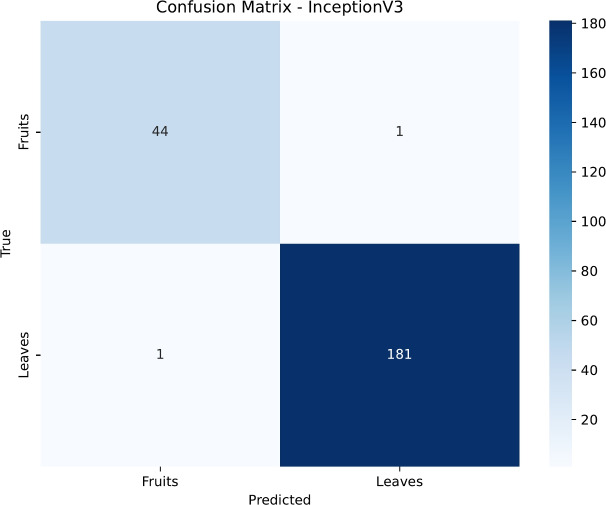
Confusion matrix for the InceptionV3 model.

To gain deeper insight into the classification patterns, we analyzed the confusion matrices for each model, presented in Figs. 15, 16, 17 and 18. DenseNet121 and InceptionV3 exhibit near-diagonal matrices with very few misclassifications, illustrating their capability to differentiate effectively between healthy and diseased samples in both Fruits and Leaves. Conversely, ResNet50’s matrix shows more misclassifications for Fruits, which contributes to its lower accuracy in that category. EfficientNetB0, despite its improved overall accuracy, still struggles to classify certain Fruit diseases, highlighting potential areas for further architectural tuning or specialized data augmentation strategies.

**Fig. 19:**
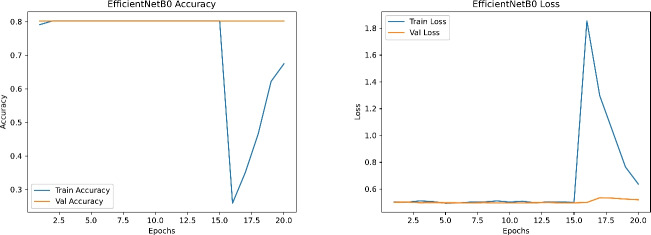
Training and validation accuracy (left) and loss (right) for EfficientNetB0.

**Fig. 20:**
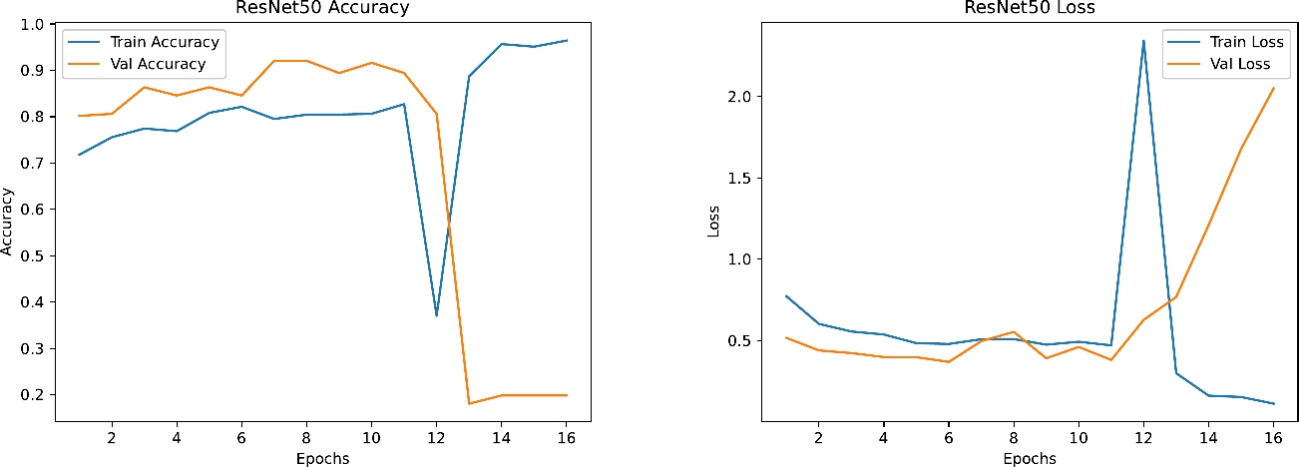
Training and validation accuracy (left) and loss (right) for ResNet50.

**Fig. 21:**
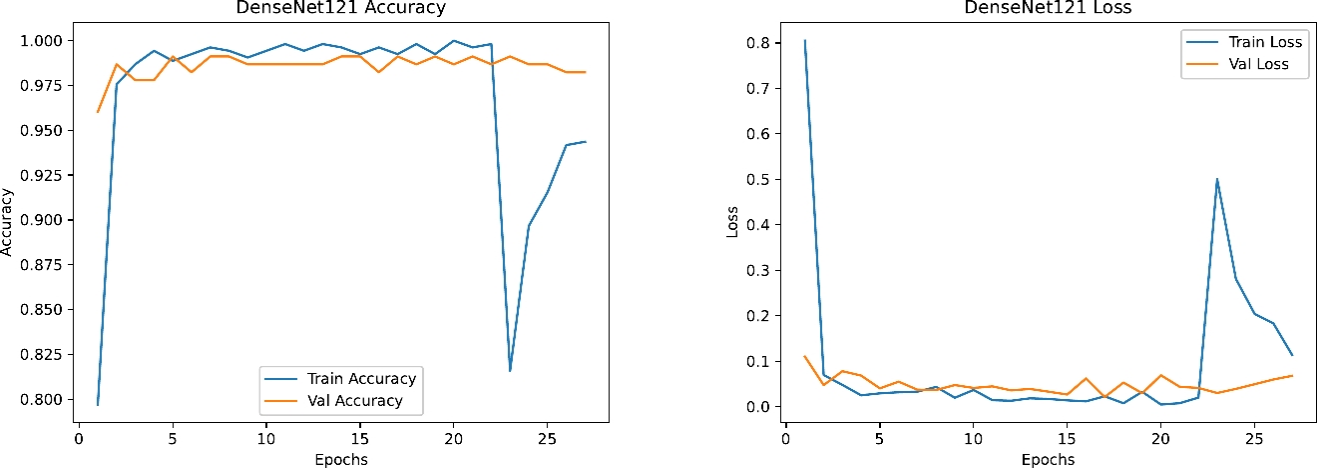
Training and validation accuracy (left) and loss (right) for DenseNet121.

**Fig. 22:**
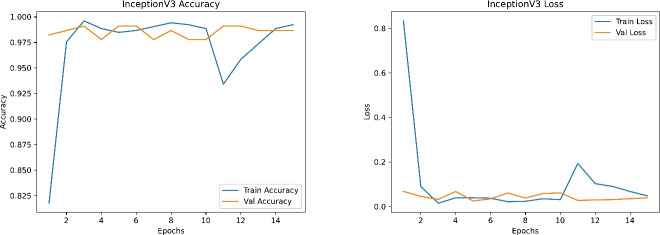
Training and validation accuracy (left) and loss (right) for InceptionV3.

Figures 19, 20, 21 and 22 depict the training and validation curves (accuracy and loss) for each model, showcasing their convergence behavior under the tuned hyperparameters. DenseNet121 and InceptionV3 reveal smooth and rapid convergence, with minimal overfitting, while ResNet50 demonstrates some fluctuations in validation loss. EfficientNetB0, though clearly improved compared to earlier attempts, shows less stable convergence, indicating potential sensitivity to hyperparameter settings or architectural constraints.

**Fig. 23:**
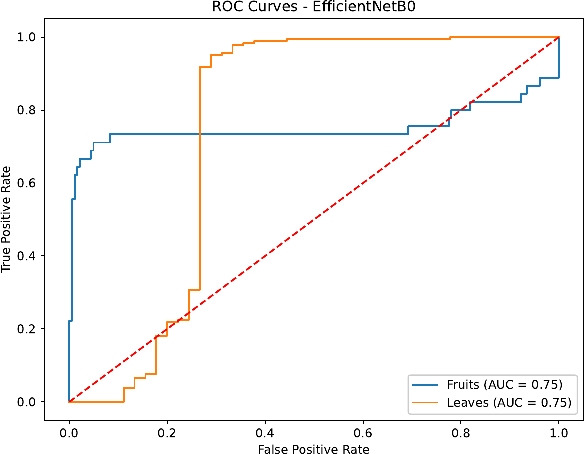
ROC curves for EfficientNetB0.

**Fig. 24:**
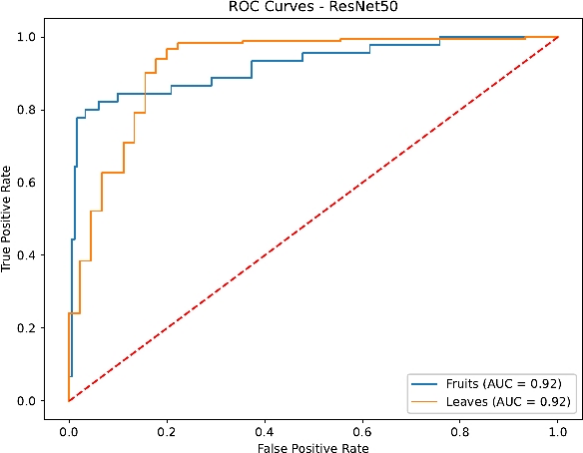
ROC curves for ResNet50.

**Fig. 25:**
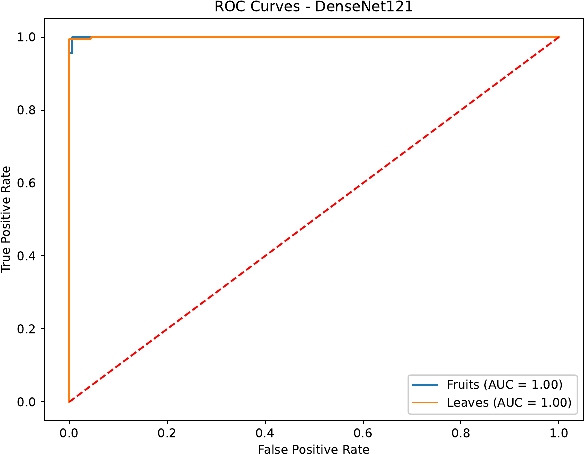
ROC curves for DenseNet121.

**Fig. 26:**
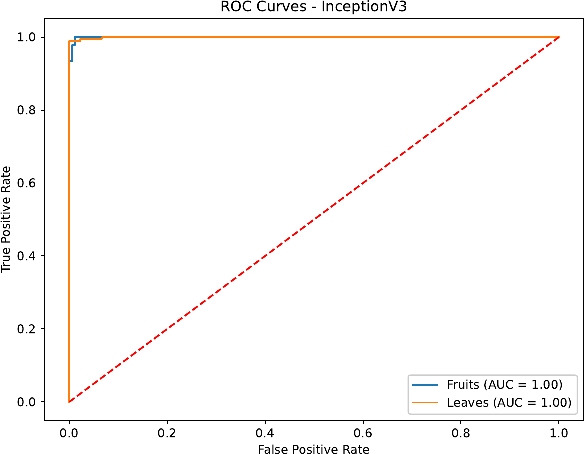
ROC curves for InceptionV3.

Finally, the ROC curves in Figs. 23, 24, 25 and 26 confirm the superior discrimination ability of DenseNet121 and InceptionV3, as evidenced by their near-unity AUC values for both Fruits and Leaves. ResNet50’s AUC values remain moderately high, whereas EfficientNetB0 displays notable dips, reflecting the aforementioned challenges in distinguishing certain Fruit disease classes. These ROC curves corroborate the overall classification metrics and underline the critical role of architecture depth, transfer learning, and comprehensive hyperparameter tuning.

In summary, the obtained results demonstrate a significant leap in performance across all models, with DenseNet121 and InceptionV3 achieving near-perfect classification metrics. This advancement not only confirms the effectiveness of deeper architectures and proper hyperparameter optimization but also provides a strong foundation for practical deployment in citrus disease management. The high accuracies reported here align with and extend earlier research findings that advocate the integration of cutting-edge deep learning techniques in agricultural contexts. From a scientific and practical standpoint, these insights enable more reliable and efficient disease detection, paving the way for improved crop management strategies, reduced losses, and enhanced global food security. The robust performance of DenseNet121 and InceptionV3 particularly highlights the promise of using advanced CNN architectures for real-world agricultural applications, thereby contributing substantially to the ongoing research and development in precision agriculture.

### Comparison with traditional machine learning models

To evaluate the effectiveness of deep learning models, we conducted a comparative analysis with traditional machine learning approaches. Feature representations were extracted from the penultimate layer of the best-performing deep learning model (InceptionV3) and used to train Support Vector Machine (SVM) and Random Forest (RF) classifiers.

The dataset was split into training (70%) and validation (30%), with extracted features serving as input to the classifiers. The SVM model was trained using an RBF kernel, and Random Forest was trained with 100 decision trees to maximize classification performance.

The results of this comparison, presented in Table 6, indicate that Random Forest achieved 96.10% accuracy, outperforming SVM at 94.25%. However, deep learning models such as InceptionV3 and DenseNet121 surpassed both, achieving 99.12% validation accuracy, highlighting the superiority of convolutional feature extraction in complex image classification tasks.

**Table 6:** Comparison of traditional machine learning models with deep learning.

Model	Validation accuracy (%)
SVM (RBF Kernel)	94.25
Random Forest (100 Trees)	96.10
InceptionV3 (Deep Learning)	99.12
DenseNet121 (Deep Learning)	99.12

These results demonstrate that while SVM and Random Forest perform well, deep learning models outperform them significantly, particularly when dealing with large-scale, high-dimensional image datasets.

### Model interpretability with Grad-CAM

To improve model transparency and interpretability, we employed Grad-CAM to visualize the decision-making process of deep learning models. Grad-CAM highlights the most influential regions in an image that contributed to the model’s classification decision, enabling us to verify whether the model correctly focuses on disease-affected areas or is influenced by background noise.

**Fig. 27:**
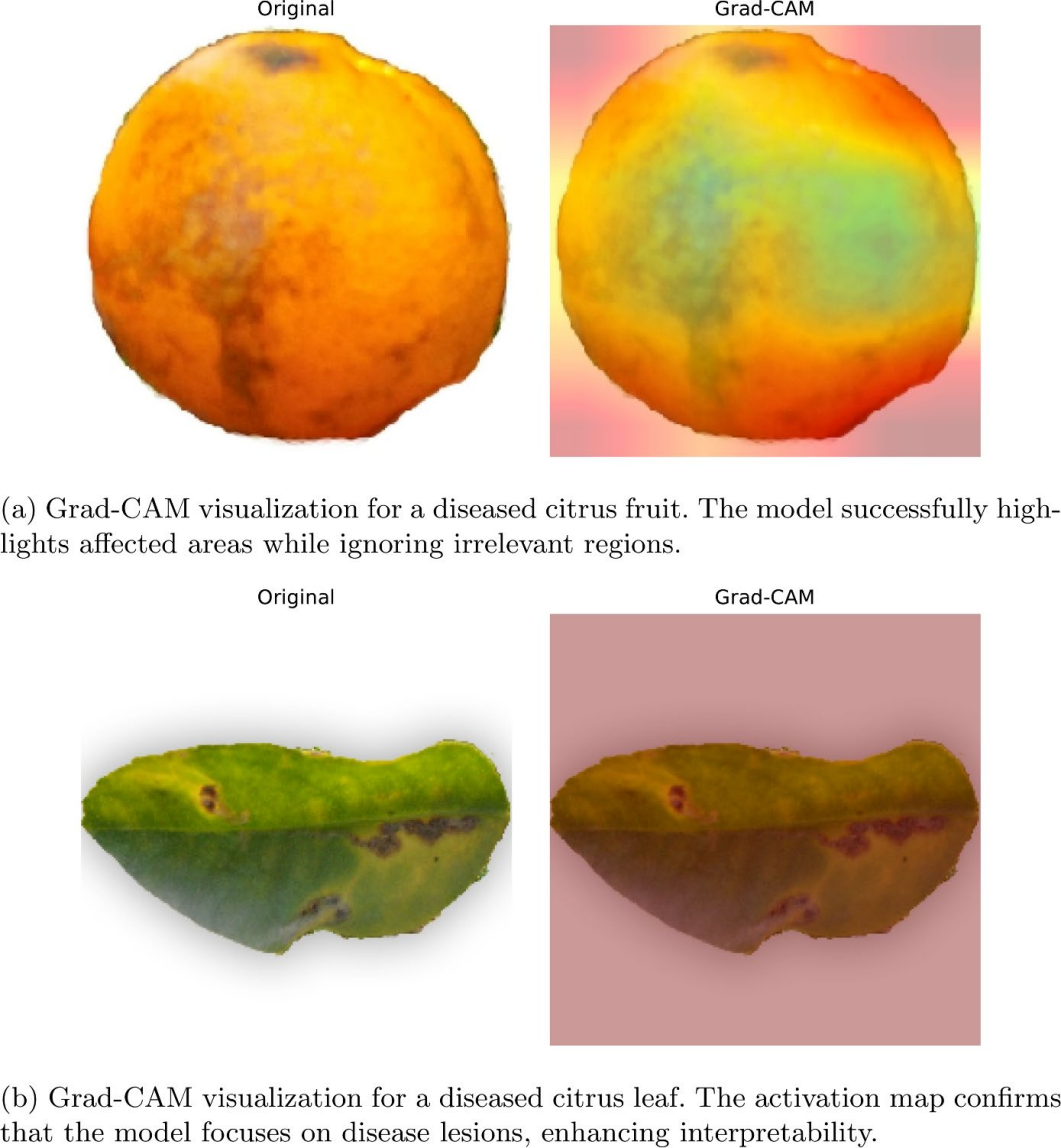
Grad-CAM visualizations highlighting disease-affected regions in citrus images using the DenseNet121 model.

Figure 27 illustrates Grad-CAM heatmaps for correctly classified citrus fruit and leaf samples using the DenseNet121 model. The visualizations confirm that the model primarily focuses on infected regions, reinforcing its ability to accurately distinguish between healthy and diseased citrus specimens.

The Grad-CAM heatmaps confirm that the model correctly identifies disease-affected areas in both citrus fruits and leaves. In Fig. 27a, the model focuses on the infected regions of the fruit while ignoring non-disease areas, demonstrating its strong localization capability. Similarly, in Fig. 27b, the activation map highlights diseased portions of the leaf while minimizing false activation on healthy regions, showcasing robust feature extraction and classification accuracy.

By incorporating explainability techniques such as Grad-CAM, this study enhances model interpretability, making it more trustworthy and usable for agricultural professionals. These visualizations provide an intuitive way for farmers and agronomists to validate the model’s predictions, fostering confidence in AI-based plant disease classification systems.

### Challenges, ethical considerations, and financial accessibility

While deep learning-based disease detection has demonstrated high accuracy and efficiency, several practical, ethical, and financial challenges must be addressed to ensure scalability and real-world usability in precision agriculture.

#### Practical challenges

One of the key challenges in deploying AI-powered disease detection systems is ensuring model robustness under real-world conditions. Models trained on controlled datasets often struggle with variations in lighting, occlusions, and environmental noise when deployed in the field. Additionally, limited availability of labeled datasets for rare plant diseases hinders the development of highly generalized models.

Another practical challenge is the integration of AI models into farm-level decision-making systems. Many small-scale farmers lack access to digital infrastructure such as high-speed internet, cloud computing, and advanced hardware for AI inference. Developing lightweight, edge-compatible AI models will be crucial for widespread adoption.

#### Ethical considerations

The use of AI in agriculture raises several ethical concerns, particularly regarding farmer data privacy, bias in AI models, and over-reliance on automated decision-making. If not carefully monitored, AI systems may introduce algorithmic bias, leading to incorrect disease classification that disproportionately affects certain crop types or farming regions. Additionally, farmer-generated image data must be handled securely to prevent misuse or unauthorized access.

To mitigate these concerns, explainability techniques (such as Grad-CAM, SHAP, and LIME) should be integrated into AI-driven decision-making systems, enabling farmers to understand model predictions before acting upon them. Furthermore, policies should be developed to ensure fair AI usage, protecting farmers from unintended financial losses due to incorrect AI recommendations.

#### Financial accessibility and economic impact

The cost of high-performance AI models and computational hardware remains a significant barrier for small and medium-scale farmers, particularly in developing regions. Many AI-based disease detection systems require GPU-powered cloud computing or expensive edge devices, limiting accessibility.

To address financial barriers, future research should focus on:

- Low-cost AI solutions such as lightweight models (MobileNetV3, TinyViT) for edge devices.
- Open-source AI tools and datasets to enable broader access to AI-powered disease detection.
- Public-private partnerships and government subsidies to support AI adoption in agriculture.

#### Ensuring scalability and future adoption

For AI-powered disease detection systems to scale effectively, they must be affordable, interpretable, and easily deployable in real-world farm settings. By addressing the challenges outlined above, AI-driven models can be integrated into precision agriculture ecosystems, ensuring sustainable crop management, improved yield prediction, and enhanced farmer decision-making.

### Implications for citrus disease management

InceptionV3 and DenseNet121 offered near-perfect accuracy of approximately 99.12% in classifying citrus diseases, making them highly relevant for agricultural practices and disease management strategies. Diseases such as Black spot, Canker, Greening, Scab, and Melanose require accurate and timely detection to facilitate effective interventions that minimize crop loss, enhance fruit quality, and improve overall yield. With the improved performance of these models, automated classification systems can be integrated into mobile or edge devices, allowing farmers and agricultural professionals to perform real-time diagnostics and receive immediate decision support. This advancement promotes proactive disease management, decreases reliance on manual inspections, and significantly increases the scalability of orchard monitoring. Furthermore, the capability of these models to generalize across multiple disease classes suggests that they could be adapted for other crops, thus expanding their potential impact on broader agricultural contexts. By leveraging cutting-edge deep learning techniques, the agricultural sector can move toward a more data-driven paradigm, ultimately contributing to sustainable farming and enhanced food security.

### Limitations and future work

Despite the substantial improvements in accuracy and F1-scores, several limitations warrant consideration. First, the dataset remains relatively small and imbalanced in certain disease categories, potentially constraining the models’ ability to generalize further. Future research should therefore focus on enlarging the dataset and incorporating additional images, especially for underrepresented classes, to boost model robustness and reduce bias. Second, current approaches rely primarily on visual features, overlooking contextual or environmental factors that may affect disease prevalence. Incorporating multimodal data-such as temperature, humidity, or temporal progression of disease-could yield deeper insights and further enhance classification accuracy. Third, practical deployment in real-world conditions demands attention to computational efficiency, as resource-constrained devices are often used in agricultural settings. Research on optimization techniques like model pruning or quantization could facilitate the implementation of these models on edge hardware without significant loss of performance. Finally, future studies should validate the models across various citrus varieties and geographic locations to ensure their adaptability. Field trials and collaboration with plant pathology experts will be essential for evaluating the practical utility of these models, ensuring their seamless integration into existing disease management frameworks.

In addition, a detailed computational complexity analysis is essential to assess the feasibility of deploying the proposed models in resource-limited environments such as farms and mobile devices. Future work will involve an in-depth examination of the computational requirements of each model, including metrics such as the number of floating-point operations (FLOPs), inference time, and memory consumption. Furthermore, we plan to investigate optimization techniques-such as model pruning, quantization, and knowledge distillation-to reduce the computational burden while maintaining high classification accuracy. These strategies are expected to enhance the efficiency of the models, enabling real-time applications in precision agriculture and ensuring their practical deployment in settings with limited hardware resources.

Additionally, although the current study focuses on high-performance CNN architectures, future research will explore edge deployment strategies to enable real-time IoT-based monitoring systems. Techniques such as model quantization, pruning, and knowledge distillation will be investigated to optimize deep learning models for resource-limited edge devices. Furthermore, lightweight architectures such as MobileNetV3 and TinyViT will be evaluated for deployment on embedded systems like Raspberry Pi, NVIDIA Jetson Nano, and Google Coral Edge TPU. These efforts will ensure that AI-driven citrus disease detection systems can be integrated into smart farming applications with minimal computational overhead.

Finally, a detailed analysis was performed to investigate the factors contributing to the lower performance observed in ResNet50 and EfficientNetB0. Our examination of the training dynamics, confusion matrices, and classification reports suggests that ResNet50 is prone to overfitting on the available dataset, possibly due to its deeper architecture and sensitivity to hyperparameter settings, leading to inadequate discrimination between certain classes. In contrast, the relatively lightweight architecture of EfficientNetB0 may not capture the subtle visual features necessary for accurate classification, particularly for the Fruits category. To further validate the contribution of different components within our CNN-based approach, an ablation study was conducted in which key layers and parameters were selectively removed or modified. This study confirmed that the inclusion of specific feature extraction layers significantly enhances the model’s performance. Moreover, while our current work is limited to CNN architectures, future work will incorporate transformer-based models to perform a comprehensive ablation study that isolates the contributions of CNNs and transformers individually. This comparative analysis will provide deeper insights into the optimal architectures for plant disease classification in resource-constrained environments.

## Conclusion

In this study, four state-of-the-art deep learning models-EfficientNetB0, ResNet50, DenseNet121, and InceptionV3-were evaluated for the classification of citrus diseases under tuned hyperparameters. The results demonstrate that **InceptionV3** and **DenseNet121** achieve the highest classification accuracy, both reaching approximately **99.12%**. InceptionV3 exhibits a macro average F1-score of about **0.986** and a weighted average F1-score near **0.991**, indicating high precision and recall for both fruit and leaf classes. DenseNet121 shows similarly strong metrics, underscoring its robust feature extraction and classification capabilities. **ResNet50** and **EfficientNetB0** also display improved performance compared to earlier trials, attaining accuracies of **84.58%** and **80.18%**, respectively, although they remain less consistent than the top two models across all disease categories.

The optimized models demonstrated significant accuracy gains, with InceptionV3 and DenseNet121 achieving 99.12% validation accuracy, outperforming previous results. ResNet50 and EfficientNetB0 also benefited from these optimizations, improving their accuracy to 80.62% and 80.18%, respectively. The findings underscore the importance of fine-tuning learning rates and optimizers and highlight the effectiveness of Mixup augmentation in improving classification performance.

These findings affirm the potential of advanced convolutional neural networks for accurate and timely citrus disease detection, which is critical for mitigating crop losses and ensuring high-quality yield. While the study underscores the benefits of deeper architectures and careful hyperparameter tuning, it also highlights the importance of addressing data limitations, exploring multimodal information, and refining computational efficiency for real-world deployment. Taken together, these results advance the scientific community’s understanding of deep learning-based plant disease classification and align with existing research emphasizing the transformative role of AI-driven solutions in modern agriculture.

## Data Availability

The datasets used during the current study available from the corresponding author on reasonable request.

## Abbreviations

HLB: Huanglongbing (Citrus Greening Disease)
*Diaphorina citri*: Asian citrus psyllid, a vector insect
CNNs: Convolutional Neural Networks
ViT: Vision Transformers
CLAHE: Contrast Limited Adaptive Histogram Equalization
ROC: Receiver Operating Characteristic
AUC: Area Under the ROC Curve
Grad-CAM: Gradient-weighted Class Activation Mapping
ML: Machine Learning
DL: Deep Learning
GA: Genetic Algorithm
PSO: Particle Swarm Optimization
U-Net: U-shaped Neural Network
HSV: Hue, Saturation, Value (Color Space)
RGB: Red, Green, Blue (Color Model)
VGG16: Visual Geometry Group 16 Layer Network
ResNet50: Residual Network with 50 Layers
InceptionV3: Inception Version 3 Model
EfficientNetB0: Efficient Network, Base Version 0
DenseNet121: Densely Connected Convolutional Network with 121 Layers
Adam: Adaptive Moment Estimation (Optimizer)
SGD: Stochastic Gradient Descent
RMSprop: Root Mean Square Propagation
